# Nanostructures as the Substrate for Single-Molecule Magnet Deposition

**DOI:** 10.3390/ijms25010052

**Published:** 2023-12-19

**Authors:** Michał Adamek, Oleksandr Pastukh, Magdalena Laskowska, Agnieszka Karczmarska, Łukasz Laskowski

**Affiliations:** Institute of Nuclear Physics, Polish Academy of Sciences, PL-31342 Krakow, Poland; michal.adamek@ifj.edu.pl (M.A.); oleksandr.pastukh@ifj.edu.pl (O.P.); lukasz.laskowski@ifj.edu.pl (Ł.L.)

**Keywords:** molecular magnetism, single-molecule magnets, nanotechnology, nanostructures, molecular engineering

## Abstract

Anchoringsingle-molecule magnets (SMMs) on the surface of nanostructures is gaining particular interest in the field of molecular magnetism. The accurate organization of SMMs on low-dimensional substrates enables controlled interactions and the possibility of individual molecules’ manipulation, paving the route for a broad range of nanotechnological applications. In this comprehensive review article, the most studied types of SMMs are presented, and the quantum-mechanical origin of their magnetic behavior is described. The nanostructured matrices were grouped and characterized to outline to the reader their relevance for subsequent compounding with SMMs. Particular attention was paid to the fact that this process must be carried out in such a way as to preserve the initial functionality and properties of the molecules. Therefore, the work also includes a discussion of issues concerning both the methods of synthesis of the systems in question as well as advanced measurement techniques of the resulting complexes. A great deal of attention was also focused on the issue of surface–molecule interaction, which can affect the magnetic properties of SMMs, causing molecular crystal field distortion or magnetic anisotropy modification, which affects quantum tunneling or magnetic hysteresis, respectively. In our opinion, the analysis of the literature carried out in this way will greatly help the reader to design SMM-nanostructure systems.

## 1. Introduction

Today’s electronics, containing components such as processing units or information storage devices, are undoubtedly reaching their technological limits. It is not an exaggeration to say that the era of silicon and bulk magnets-based electronic equipment is slowly but surely coming to an end due to the physical limitations of these materials. For example, the magnetic domain size, serving as a single bit, seems to be an obstacle that is impossible to overcome by means of classical physics and electronics as we know it [1]. In addition, the applicability of a magnetic structure is constrained by its material limitations, such as being ferromagnetic and maintaining its magnetic orientation under typical conditions for an extended period. Nevertheless, the need for the minimalization of devices is still strong, with the simultaneous need for enlarging their memory, capabilities, and speed of operation. One of the most promising strategies to address these challenges is to ’look deep down’ and research the area of molecular magnetism.

Single-molecule magnets (SMMs, a term first used in 1996 [2]) are a wide class of metal–organic compounds whose single, separate molecules at low temperatures can exhibit characteristics similar to those of bulk magnets. Since the first reports on magnetism in the archetypical Mn${}_{12}$-ac compound were made in 1991 [3], they have been gaining increasing worldwide interest among chemists, physicists, crystallographers, and material scientists. There are a couple of main features that, on the one hand, define them and, on the other hand, make SMMs so interesting and prospective: the large spin of the molecule in its ground state and strong magnetic anisotropy; the slow relaxation of magnetization and open-loop magnetic hysteresis below a certain blocking temperature, T_B_; and the coexistence of classical and quantum effects [4,5]. The blocking temperature is one of the key features describing SMMs as it tells what the maximum temperature can be for a given SMM to be operative. T${}_{B}$ is essentially the temperature below which the magnetic moments of the SMMs become “frozen” or blocked in a particular orientation due to the energy barrier between different magnetic states. Above this temperature, thermal energy becomes sufficient to overcome the barrier, allowing the magnetic moments to undergo spontaneous fluctuations. To date, the record for the highest T${}_{B}$ of an SMM is assigned to the dysprosium metallocene cation [(Cp^iPr5^)Dy(Cp*)]^+^ (Cp^iPr5^-penta-iso-propylcyclopentadienyl, Cp*-pentamethylcyclopentadienyl), for which the value of 80 K has been reported [6]. Nevertheless, we cite this example here only for illustrative purposes, as, to the best of our knowledge, examples of high-temperature metallocene dysprosium single-molecule magnets anchored onto nanostructures have not yet been documented. The most important point, however, is that the abovementioned properties of SMMs appear, due to the inner chemical structure of each molecule, without any cooperative effects and in the absence of short- or long-range intercluster interactions [7]. The combination of the aforementioned properties along with understanding and learning to control them open the door to wide applications and brand new technologies based on molecular nanomagnets, such as quantum computing and spintronics [8,9,10].

The key point of molecular electronics realization relies on the ability to address the magnetization of each individual molecule [11]. Therefore, to really make use of all of the wonders of molecular magnets, put them to work, and start creating devices that utilize them, one obviously needs to place them on the surface. There is a need for some kind of a matrix or basis onto which they can be anchored, preferably in a controlled and precise manner. Of course, depositing single molecules on surfaces is not a trivial task, and there are a lot of issues that should be resolved during the process. Many attempts have been made in this direction so far; however, the preservation of initial molecules’ functionality and properties upon surface deposition is still a challenging task [12]. The surface–molecule contact may cause additional factors that could affect the magnetic performance of molecules, such as reduced intermolecular forces and the appearance of surface–molecule interactions, possible molecular crystal field distortion, or magnetic anisotropy modification affecting quantum tunneling or magnetic hysteresis [13].

Depending on the type of SMM, the type of functionalized surface must also be wisely chosen. The significant progress in molecular electronics has lead to a growing fascination with assembling nanodevices and circuits directly from molecular constituents (self-assembly methods) [14,15], in particular, molecular spintronics, the foundation of which is the electron’s intrinsic spin and associated magnetic moment, in addition to its fundamental electron charge, and which, simply put, focuses on the injection, manipulation, and detection of spins requires special solutions . For example, such applications as spin valves, molecular transistors, or qubits demand a single layer of molecules or even a few molecules per device [16,17]. The realization of such nanodevices therefore leads to the necessity for the organization of SMMs onto low-dimensional substrates (e.g., nanostructures—systems with nanometric sizes in at least one dimension), where molecules can be individually manipulated and investigated. Therefore, the anchoring of SMMs on the surface of the nanostructures is gaining much attention in the field of molecular magnetism and should be investigated more deeply. What also makes this idea so accurate is the large specific surface area to overall volume ratio of nanostructures. It is shown that, for a given sample volume, shrinking down the size of structures having a given shape (i.e., spherical or cubic) will result in a great increase in the total specific surface area of particles comprising that sample. The more surface area there is, the more space there is for magnetic molecules to be placed on. Also, explicit size compatibility exists between single-molecule magnets (which mostly do not exceed 50 nm in diameter) and nanostructures * which are also nano-sized but large enough to provide an appropriate basis for molecular magnets). This compatibility not only enhances the functionality and versatility of the nanostructures but also opens up new avenues for exploring phenomena at the intersection of molecular and solid-state magnetism.

As mentioned earlier, the most efficient way to build nanodevices is the self-assembly method [18,19] This procedure can also be used to deposit SMMs on some specific surface precisely. Undoubtedly, in the process of adapting molecular magnets for deposition, solubility plays a key role. It determines whether the SMMs can be dispersed evenly in solution and effectively incorporated into the nanostructure. The low solubility of SMM-containing crystals can lead to the formation of bulk agglomerates of magnetic molecules, which are unable to be patterned or to create uniform, monodisperse layers. In general, not all classes of SMMs are highly soluble in common solvents, which makes it difficult to achieve the desired level of dispersion; therefore, various attempts have been made to overcome this issue. The main approach involves attaching to magnetic molecules functional groups that can interact with the solvent and increase their solubility. For example, carboxylates, such as stearate groups, can be added to SMMs to improve their solubility in organic solvents [20,21], or *tert*-butyl (pivalate) groups, to improve solubility in nonpolar solvents [22]. It is important to note that the solubility of nanomagnets can also be affected by their size and shape. In general, smaller SMMs tend to be more soluble than larger ones, and SMMs with more compact structures are more soluble than those with more extended structures [23]. In this paper, we will outline how challenging yet necessary it is to tune the solubility of SMMs, mainly by ligand-exchange reactions, and how significantly such reactions can alter the properties of single-molecule magnets [24].

Summarizing and presenting recent scientific developments in the field of depositing SMMs on nanostructures will be the scope of this review article. First, we will focus on magnetic molecules themselves. We will briefly describe the quantum mechanical origins of their magnetic behavior and present the most studied types of SMMs. For the purpose of this review article, we have chosen the archetypical Mn_12_ molecules, iron-based Fe_4_ and Fe_8_ family, chromium-including heterometallic Cr_7_X rings, and single-ionic LnPc_2_ lanthanoid phthalocyanines, mainly because of their representativeness, emerging from their abundance in the field of molecular magnetism. After that, we will introduce the most commonly researched nanostructures that may serve as substrates for nanomagnets, along with their most important properties, and outline their relevance for compositing them with SMMs. This will include nanoparticles, metallic thin films, carbon-based nanostructures (graphene, fullerenes, and nanotubes), metal–organic frameworks, and porous silicas grouped into categories based on the dimensionality of structures. Next, we will have a look at particular literature examples of functionalization nanostructures with single-molecule magnets. In this case, we will divide the examples in terms of the material of the substrates rather than their dimensionality. Subsequently, we will move on to outlining the advantages that the functionalization processes bring and reviewing some of the possible applications of these novel composite materials comprising single-molecule magnets and nanometric objects. The article will eventually be wrapped up with a brief summary of its contents.

## 2. Characteristics and Main Representatives of SMMs

Prior to discussing the deposition of magnetic molecules on particular nanostructures, it would be beneficial to provide a brief overview of the synthesis, molecular structure, and origin of the distinct magnetic properties exhibited by commonly investigated single-molecule magnets. In this section, we will concentrate on the main chemical classes of SMMs that are conducive to surface deposition, while also examining their magnetic properties and physicochemical characteristics, starting from the archetypal dodecamanganese (Mn${}_{12}$) molecule. Then, we will describe iron-based (Fe_4_) and chromium-based (Cr_7_X) SMMs, and we will end with a description of lanthanoid phthalocyanines (LnPc_2_) single-ion magnets (SIMs). Furthermore, we will demonstrate how feasible it is to alter the outer organic shell of these SMMs to incorporate various functionalities (such as anchoring capabilities) and possible altering of molecules’ magnetic properties.

### 2.1. The Mn_12_ Family

When it comes to introducing particular single-molecule magnets, it is reasonable to start with the archetypal example of the Mn_12_-ac molecule. The full chemical formula of this compound is [Mn_12_O_12_(CH_3_COO)_16_(H_2_O)_4_]·2CH_3_COOH·4H_2_O (**1**), and it was first synthesized in 1980 by Polish crystallographer Tadeusz Lis via the addition of permanganate to a solution of manganese acetate tetrahydrate [25]. Although purely molecular magnetism was not known yet at that time, in his work Lis accurately pointed out that ‘(…)such a complicated dodecameric unit should have interesting magnetic properties’. Indeed, research done in the early 90’s showed that these molecules exhibit not only large total spin in the ground state but also a magnetic hysteresis (thus, a magnetization in zero field), even when they are placed far apart from each other, that is, when collective long-range interactions are excluded [3,5,26,27].

**Figure 1:**
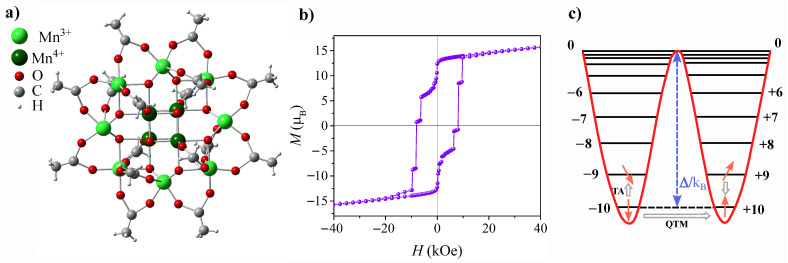
Representation of Mn${}_{12}$-ac molecule crystal structure (top view) (**a**), hysteresis loop measured at T = 2.0 K presenting characteristic steps at the resonance fields (**b**), and schematic representation of the energy level splitting for an S_T_ = 10 ground state with the anisotropy barrier Δ/k_B_ for spin reorientation (**c**). Grey arrows indicate thermally assisted (TA) and quantum tunneling (QTM) mechanisms of spin reversal.

Mn_12_-ac molecule is illustrated in Figure 1a. It has a core-shell structure, with a core comprising twelve manganese ions, hence the name: eight outer Mn^3+^ ions with integer spins S = 2, and four inner Mn^4+^ ions with half-integer spins S = 3/2, connected by bridging oxygen atoms [28]. Taking into consideration that Mn^3+^ and Mn^4+^ are coupled antiferromagnetically by a superexchange interaction, and following the straightforward formula for a total spin of a molecule’s core: S_T_ = S_Mn3+_ + S_Mn4+_, one can easily evaluate that S_T_ = 10. The suffix ‘ac’ stands for acetate as the shell of the molecule comprises acetate ligand groups. These long, magnetically neutral acetate chains provide spacing between the Mn_12_-ac magnetic cores, which helps to eliminate long-range interactions between them. In addition to the high spin of the ground state, the distinctive feature, which determines SMMs magnetic behavior, is easy-axis anisotropy, which in Mn${}_{12}$ is caused by the elongation of the Mn^3+^ ion axes (Jahn–Teller distortion). Due to large anisotropy, zero-field splitting (ZFS) of the S_T_ = 10 state provides the appearance of 10 doublets, with extremes being m_s_ = ±10 [26]. Between two stable states of m_s_ = ±10, an energy barrier exists, which for the Mn${}_{12}$-ac molecule is equal to ∼65K [5]. Overcoming the barrier in the relaxation process is made possible by the coupling of the spin system to the environment (i.e., spin–phonon coupling). As a result, at sufficiently low temperatures the relaxation becomes so long that magnetic hysteresis can be observed. The distinguishing characteristic of molecular magnets within the realm of magnetic systems, however, lies in the coexistence of classical and quantum magnetization reversal mechanisms. In addition to the thermally activated process described above, spin reversal in molecular magnetics can also occur via the quantum tunneling of magnetization (QTM) [29], which involves either the ground doublet or thermally excited states (TAQTM) [30]. When the quantum admixing of the “up” and “down” states occurs, a direct transition under the barrier is observed, leading to the appearance of distinct steps in the hysteresis loop. The characteristic hysteresis loop and energy levels of Mn${}_{12}$-ac are depicted in Figure 1b and Figure 1c, respectively. Note, that the observed hysteretic behavior of individual molecules, as well as the coexistence of classical and quantum mechanical spin reversal mechanisms, distinguishes SMMs from other magnetic systems.

Despite the outstanding magnetic properties of the prototypical, acetate version of the Mn${}_{12}$ single-molecule magnet even in its original state, it is unfortunately not free of flaws. For example, the bulk, crystalline form of Mn_12_-ac is fairly stable under atmospheric pressure and room temperature, but it decomposes easily in CH_3_CN solution exposed in the air. This is due to the redox reactions accelerated by oxygen and water contained in the air [31]. Furthermore, the poor solubility of the Mn_12_-ac compound in organic solvents poses a challenge in selecting suitable solvents for the formation of thin films and in conducting electrochemical studies across a range of solvents.

Bearing the need to adapt nanomagnets to practical applications in mind, it is reasonable to now introduce other derivatives of Mn_12_, which are suitable for binding the molecule to the specific surface. Talking about the modification of Mn_12_-ac, due to high reactivity and chemical adaptability its cluster easily engages in ligand-exchange reactions when combined with the proper carboxylate or bidentate ligands without causing molecular disruption. From now on, we will depict the general Mn_12_ complex formula as [Mn_12_O_12_(O_2_CR)_16_(H_2_O)_4_], with R being an appropriate ligand group (i.e., for Mn_12_-ac R = CH_3_COO^−^). Substituting acetate ligands for other organic groups implies altering many chemical properties of the molecule: it can enhance its solubility in organic solvents [31], modify redox potentials [32], or change the direction of the shape or magnetic anisotropy [33]. Also, applying alternative ligands can affect nanomagnets’ intramolecular spin exchange interactions [34] or be used to provide better SMM-surface bridging [35,36].

Numerous analogues of the Mn${}_{12}$ molecule have been successfully synthesized to date, showing a significant range of variations in the structure of the ligands of the molecules. Nevertheless, it is necessary to outline the ligand-exchange reaction mechanism and give a couple of qualitative examples. One can divide ligand exchanging, putting aside the site-selective substitution case, into two cases (carboxylate and non-carboxylate substitution), but, in general, the reaction scheme looks like the following:

$$\begin{array}{c} \left[Mn_{12}O_{12}(O_{2}CMe{)}_{16}(H_{2}O{)}_{4}\right]+16RCO_{2}H⇆\\ \;\;\;\;\;\;\;\;\;(16-x)MeCO_{2}H+[Mn_{12}O_{12}(O_{2}CMe{)}_{x}(O_{2}CR{)}_{16-x}(H_{2}O{)}_{4}] \end{array}$$

The reaction given above is reversible; therefore, additional treatments are required to complete the reaction, such as (a) using an excess of RCO_2_H, which is more acidic than MeCO_2_H, and/or (b) eliminating MeCO_2_H by its toluene azeotrope, to shift the equilibrium of the reaction to the right and ensure a high yield of pure product for various carboxylate types. Method (b) is particularly useful for adding carboxylate groups with an acid dissociation constant comparable to or higher than that of acetic acid. It is worth noting that the Mn_12_ complex is not necessarily required as the starting material for the carboxylate substitution [20]. Different derivatives of **1** with various carboxylates can be obtained by treating a slurry of the acetate derivative with the desired carboxylic acid in a suitable solvent. The substitution of acetate with carboxylic acid can be achieved by exploiting the different acidity of the carboxylic acid, which shifts the equilibrium towards the production of the weakest acid. The carboxylate groups in the axial position are more susceptible to substitution by less basic incoming ligands than their equatorial counterparts [37], although the synthesis of mixed-ligand derivatives often leads to partial substitution of both axial and equatorial positions [38]. For example, the preparation of the benzoate derivative involves treating **1** with a 100% excess of benzoic acid in CH_2_Cl_2_, which leads to the majority of the CH_3_COO^-^ groups being exchanged but not all [27]. The obtained black solid contains both acetate and benzoate, which can be further treated with a tenfold excess of benzoic acid to give the completely substituted derivative. Another approach for the substitution reaction employs the [Mn_12_O_12_ (tBu-COO)_16_(H_2_O)_4_] derivative, which has a tert-butyl group (-C(CH_3_)_3_) that enhances the solubility of the cluster in organic solvents [39]. Also, it is worth noting that not all Mn_12_ derivatives exhibit an S_T_ = 10 ground state as the competition of antiferromagnetic interactions can stabilize smaller spin states such as S_T_ = 9 with minor changes of their relative strength [40]. On another note, there are other manganese-based SMMs, differing in nuclearity, showcasing the spin ground state exceeding the value of 10. Especially, the Mn${}_{6}$ species are worth mentioning here. The S${}_{T}$ value of Mn${}_{6}$ compound derivatives may vary from 4 up to 12 [41,42]. Moreover, these molecules demonstrated remarkable versatility in incorporating diverse organic ligands, rendering them promising and well-suited for surface deposition [41,43,44].

**Figure 2:**
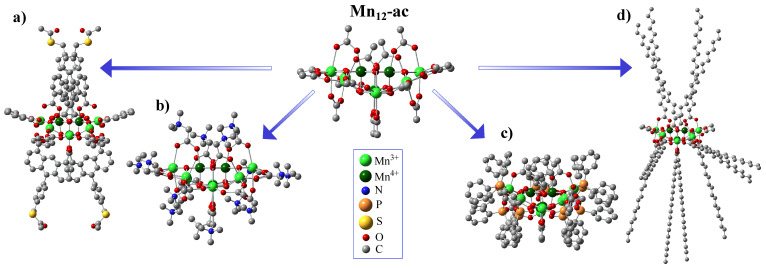
Representation of Mn${}_{12}$-ac structure and its possible derivatives obtained through ligand-exchange reaction: Mn${}_{12}$-benzoate-ADC (**a**), Mn${}_{12}$–betHPF_6_ (**b**), Mn${}_{12}$-phn (**c**), and Mn${}_{12}$-stearate (**d**).

The substitution of carboxylate groups has been widely utilized in Mn_12_ chemistry. Searching for a way to enhance the potential of the Mn_12_ cluster for information technology applications, Pacchioni et al. have developed a new route for the site-specific functionalization of the initial molecule [45]. As it was shown, applying anthracene-1,8-dicarboxylic acid (H${}_{2}$ADC) for the site-specific ligand replacement provides the exceptional anisotropic shape of the molecule (see Figure 2a), giving a straightforward route for the deposition of SMMs with easy axes perpendicular to the surface. Zhao et al. prepared the CF_3_CO_2_^−^ derivative as a potentially better candidate for incorporating Mn_12_ into films due to its high solubility and volatility [46]. Gerbier et al. utilized this approach by introducing spin-carrying carboxylate groups onto the Mn_12_ molecule using 1-[N-t-butyl-N-(oxyl)amino]-4-benzoic acid radicals, which coupled antiferromagnetically to the [Mn_12_O_12_] core [39]. Bian et al. prepared naphthalene-carboxylate derivatives that assembled two-dimensionally via π-π interactions [47], and Willemin et al. made methacrylate derivatives to produce chemically and thermally stable nanocomposites [48]. Coronado et al. have reported the way for the synthesis of the polycationic form of Mn${}_{12}$ with an S_T_ = 11 ground state using betaine hexafluorophosphate salt (see Figure 2b) [49]. The authors report that a variety of crystalline SMM salts might be made by this straightforward metathesis process, and it serves as a helpful precursor for the organization of these magnetically active substances on metal and metal oxide surfaces. Fonin et al. reported the using of diphenylphosphinate ligands in the Mn${}_{12}$ molecule (see Figure 2c) to provide its surface deposition on the gold surface and analyze the method of surface orientation of such SMMs [33]. Park and Jeong replaced acetate groups with stearate groups to prepare a highly hydrophobic Mn_12_-st derivative (see Figure 2d), to make it much more soluble in a majority of organic solvents [31]. Such modified SMMs were shown to be successfully applied for the precise functionalization of the silica surface [50].

### 2.2. Iron-Based SMMs—The Fe_4_ Family

**Figure 3:**
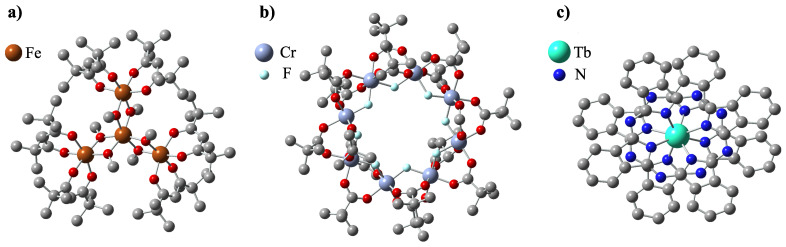
Representative examples of the crystal structure of SMMs favorable for surface deposition: Fe${}_{4}$ (**a**), Cr${}_{8}$F${}_{8}$ (**b**), and TbPc${}_{2}$ (**c**).

Soon after the discovery of single-molecular magnetism in Mn_12_ units, the search began for other molecules with similar properties. The scientific community had not had to wait long for the progress in that matter as the new ‘stars’ shone among the rapidly growing class of nanomagnets. The ‘stars’ were, in fact, Fe_4_ tetrairon complexes, reported to behave as single-molecule magnets in 1999 by Barra et al. [51]. The chemical formula of the archetypal compound for this class is [Fe_4_(CH_3_O)_6_(dpm)_6_] (**2**), with Hdpm standing for dipivaloylmethane: (CH_3_)_3_CCOCH_2_COC(CH_3_)_3_. In jargon, they quickly gained their nickname ‘iron-stars’ because of the star-like configuration of four Fe^3+^ ions comprising the magnetic core of the molecule. As seen in Figure 3a, one of the iron ions is placed in the center and is connected to the other three by alkoxide bridging bonds. There are six methoxide groups surrounding the tetrairon magnetic core, and six dipivaloylmethane groups constituting the outer ligand shell of the molecule.

Magnetic features of Fe_4_ are analogous to those of Mn_12_-ac and are fully sufficient to be classified as single-molecule magnets. In the neutral state, as mentioned previously, the magnetic core consists of four Fe^3+^ ions (S = 5/2), where antiferromagnetic interaction between the central iron ion and the peripheral ones is mediated by the methoxo bridges and leads to a ground-state spin of molecule S_T_ = 5. The molecule displays a strong uniaxial anisotropy, which originates from the asymmetry of the molecule and lifts the degeneracy of the spin ground state into five doublets and a singlet distributed over a spin reversal energy barrier. Finally, but not least importantly, Fe_4_ compounds, just like Mn_12_’s, exhibit slow relaxation of magnetization when thermal energy is sufficiently low (lower than blocking temperature *T*_B_), and a characteristic, stair-like hysteresis loop, which is a direct result of the quantization of the molecule’s total magnetic moment and the presence of the energy barrier Δ/k_B_ between its two stable states. For this molecule, slow magnetic relaxation was observed below 1 K, which is in agreement with the relatively small ZFS parameter, −0.20 cm^−1^, and the barrier for magnetic reorientation of 7 K [51].

Although the discovery of magnetism ’inside’ iron-based complexes was not an improvement relative to Mn_12_ in terms of either the ground state spin value or the ZFS parameter, it quickly became relevant in the field by raising hopes for addressing some of the flaws of the latter, mainly the molecules’ robustness and suitability for functionalization [12].

Just like with the Mn_12_ case, Fe_4_ comes with numerous different derivatives, varying not only in the type of organic ligand groups but also in the nuclearity (number and valence of Fe ions) comprising the molecules’ magnetic core. In fact, a consensus exists in the scientific community that the first iron-based complex to exhibit single-molecular magnetic behavior was actually the [Fe_8_O_2_(OH)_12_(tacn)_6_]^8+^ (**3**) (tacn = 1,4,7-triazacyclononane) complex cation [52,53], which has eight core Fe^3+^ ions, making it an octanuclear cluster, more appropriate to shortly denote as Fe_8_. It is worth pointing out that the synthesis route of this compound (the reaction of [Fe(tacn)Cl_3_] with NaBr in H_2_O) was also reported long before its superparamagnetic properties were revealed [54].

In **3**, two oxides and four hydroxo bridges coordinate the two internal Fe^3+^ ions (denoted as Fe1 and Fe2) in an octahedral arrangement. Two lateral Fe3 and Fe4 ions are attached to three nitrogen atoms of the tacn molecules, two hydroxides, and one oxide ion. On the other hand, there are four apical (Fe5, Fe6, Fe7, and Fe8) ions Fe^3+^ coordinates to three nitrogen atoms and three hydroxides (for details see Figure 1 at [55]). The presence of three distinct Fe sites is confirmed by Mössbauer spectroscopy [56]. The oxides create $\mu_{3}$ bridges, while the hydroxide ligands form $\mu_{2}$ bridges. The structure formed by Fe1, Fe2, Fe3, and Fe4 is a common configuration in polynuclear metal complexes, known as the ‘butterfly structure’. This structure causes spin frustration effects, which make it challenging to predict the most stable spin arrangement from a magnetic standpoint. Nevertheless, the ground state total spin of the molecule was determined to be S_T_ = 10 by means of the theoretical investigation of the Hamiltonian matrix and magnetic susceptibility with the use of irreducible tensor operators (ITO) approach [57].

Fe_8_ was the first compound after Mn_12_-ac to be reported exhibiting molecular-origin magnetic hysteresis [58]. It was also the first compound in which the physics of the QTM was extensively studied, providing a detailed understanding of the mechanisms underlying these processes [59,60,61].

The iron-based SMM family is not as voluminous as its manganese-based counterpart; nevertheless, it is still numerous. We will now indicate a couple of the most interesting and representative examples of its ‘members’.

The [Fe_22_O_14_(OH)_3_(O_2_CMe)_21_(mda)_6_](ClO_4_)2·4H_2_O·4EtOH·4Et_2_O (**4**) (mda— malondialdehyde, CH_2_(CHO)_2_) marks the largest nuclearity member of the family, with its core cubane consisting of 22 Fe^3+^ ions. Despite high nuclearity, the ground state spin value was reported to be S_T_ = 0, due to the configuration of Fe^3+^ ions, leading to antiferromagnetic coupling between them. Complex **4** has demonstrated solubility in multiple organic solvents and has undergone many reactivity studies. Among these studies were experiments involving the use of bases to test if the deprotonation of the bridging hydroxide ions could induce a change in nuclearity. However, in cases where a pure product was successfully isolated, it was found that this complex remained unchanged. Additionally, various alterations to reaction ratios and solvents were attempted, but **4** remained the sole product identified. Based on these findings, it can be concluded that this is a highly thermodynamically stable compound [62].

New iron-based SMMs are also obtained by ligand substitution processes. For instance, by exchanging methoxide groups from **2** for tripodal L^1^: 2,2-bis-(hydroxymethyl)-10-undecen-1-ol alkenyl group and L^2^: 11-(acetylthio)-2,2-bis(hydroxymethyl)undecan-1-ol thioacetyl group, it was possible to create two new derivatives of **2** (Fe_4_(L^1^)_2_(dpm)_6_ and Fe_4_(L^2^)_2_(dpm)_6_) with a ‘wire-like’ geometry [63]. Such tripodal groups possess the η^2^, η^2^, η^2^, μ^4^ bridging mode, which implies great structural and chemical stability, eventually making magnetic molecules suitable for deposition onto technologically important substrates [64,65,66]. The synthesis of such compounds was executed starting with primary [Fe_4_(CH_3_O)_6_(D_18_)(dpm)_6_] dissolved in dry ethanol, which leads to progressive decomposition of the SMM, leaving sole Fe(dpm) moieties [67]. To this solution, L^1^ alkenyl and L^2^ thioacetyl groups were respectively added, and thanks to the geometry of these molecules site-specific substitution occurred, leading to the creation of Fe_4_ analogs, consisting of an iron magnetic core and two chain-like ligands on its opposite sides. Crafting these molecules fulfilled the need for having SMMs with functionalities enabling the creation of metal-molecule-metal junctions, and by means of DC and AC magnetometry, as well as HF-EPR, it was confirmed that magnetic properties of archetypal **2** complex were retained. Similar propeller-like derivatives of **2** maintaining their magnetism were reported in 2008 when the ligand exchange procedure was applied to substitute methoxide groups for chain-like polymeric groups of 11-(acetylthio)-2,2-bis(hydroxymethyl)undecan-1-ol [68].

It is also worth noticing that ligand replacement is carried out not only for the purposes of enhancing SMMs stability or ability for deposition on given surfaces but may also serve as the method for increasing molecules’ magnetic anisotropy constant **D** [67].

In addition to the above-mentioned most prominent examples of varieties of iron-based molecular magnets, other mentionable examples are: [Fe_4_(acac)_6_(Br-mp)_2_] [69], Fe_4_C_3-5_SAc [70], [Fe_4_(Ph–C (CH_2_O)_3_)_2_(dpm)_6_] [71], or [Fe_4_(PhpPy)_2_(dpm)_6_] [72]. Quite a few more can be found in the literature [12,73,74].

### 2.3. Chromium-Based Rings

The next relevant, widely studied class of SMMs comprises derivatives of the compound [Cr_8_F_8_(^t^BuCOO)_16_] **5**, having in its center a regular, planar octagon comprising eight antiferromagnetically coupled Cr^3+^ ions, bridged by fluorine(I) ions [75], surrounded by sixteen ^t^BuCOO groups. The crystal structure of **5** is shown in Figure 3b. Since one of the chromium ions is very often replaced with nickel, this class is usually referred to as Cr_7_Ni, with the general formula being (R_2_NH_2_)[Cr_7_NiF_8_(RCOO)_16_]. Although the ground spin state of **5** is S_T_ = 0, substitution for one divalent Ni^2+^ ion necessitates increasing this value to 1/2 [76,77,78]. As a result of this procedure, a ground state doublet may be formed with an energetic separation from higher energy levels. This creates a level structure that is suitable for qubit implementation, which is why this type of nanomagnet is particularly studied for applications in quantum computing [79,80].

The replacement of one of the Cr(III) ions may apply not only to Ni(II) but also to many different divalent metals, such as Zn(II), Fe(II), Co(II), Mn(II), or Cd(II) [79,81]. Of course, depending on the ion being put in the place of chromium, one can tune the total ground state spin of the molecule, but reaching higher and higher S_T_ values seems not to be the case with Cr_7_X complexes as their value lies in somewhat different aspects.

Besides the aforementioned quantum computing applications, this type of system is of particular interest due to two factors: the possibilities for investigating the origins of magnetic anisotropy, as they form a highly symmetrical series of complexes [75,78], and their great chemical versatility, stability, and ease of modification, which, of course, makes them suitable and flexible for accommodation on surfaces [82,83].

The collective symmetry of Cr_7_X compounds is often imposed by crystallography but can also be idealized. Magnetic anisotropy can be observed in the response of ions in a molecule to an external field (known as g value anisotropy), as well as in the splitting of a spin state manifold in the absence of a magnetic field. The total magnetic anisotropy of clusters is determined by several factors, including the sum of the single-ion anisotropies of the transition metal ions, the dipolar interaction between ions, and the anisotropic exchange interaction between ions, which relies on the admixture of excited states into the ground state by spin–orbit coupling (SOC) [75].

On the other hand, in the case of Cr_7_X, one can not only exchange the Cr^3+^ core ion to adjust magnetic properties, or substitute [^t^BuCOO] ligands for other organic groups to, for example, enhance their solubility, but such heterometallic rings can be also used as cages for other, smaller molecules [77,83,84].

While ending this section, it is really worth underlining two recent reports demonstrating the versatility and ability to create molecular architecture towards quantum computing using the narrow field of heterometallic rings science. McInnes, along with coworkers, published a broad and fascinating study showing that such molecular wheels not only can exhibit magnetic phenomena never seen before but, due to their chemical versatility, may serve as ‘building blocks’ for larger, supramolecular structures [81]. In this work, they have shown how derivatives of Cr_7_Ni rings can be modified and used to build horseshoe structures and their complexes, as well as how they can be used as Lewis acids, as components of hybrid rotaxanes, or ligands surrounding other molecules, for constructing larger, more sophisticated units. In 2022, Lockyer et al. took a Cr_7_Ni-based, five-spin supramolecule, characterized it, and determined interaction energies between the spins by means of electron paramagnetic resonance (EPR). Using the measured parameters, the researchers suggest that this specifically designed supramolecule could be utilized to simulate quantum decoherence in maximally entangled Bell states, which could potentially be employed in quantum teleportation [85].

### 2.4. Lanthanoid Phthalocyanines

The last kind of single-molecule magnets we decided to review are lanthanoid phthalocyanine systems—quite a new and promising class of SMMs. Their general chemical formula is [Ln(III)Pc]^n^, where *n* is the charge of the compound; Pc is short for phthalocyanine C_32_H_18_N_8_ groups; and Ln(III) is one of the lanthanide elements, typically Tb^3+^ or Dy^3+^. They are often called ‘double-deckers’ because of their structure—they consist of one lanthanoid ion sandwiched between two flat phthalocyanine ligands, as seen in Figure 3c. Pc groups are rotated by 45${}^{\circ }$ relative to each other. The Ln ion is held in the middle via TBA^+^ (N(C_4_H_9_)_4_^+^) bridging groups. In contrast to previously described classes of nanomagnets, these compounds contain only one magnetic center (the Ln ion), so sometimes they are referred to as SIMs (Single-Ion Magnets) [86]. In 1996, Koike et al. provided an extensive X-ray study on the crystallography of such compounds [87]. However, it was not until 2003 that they were first reported to exhibit slow magnetization relaxation by Ishikawa et al., when they published the results of detailed AC susceptibility measurements, thus confirming the purely molecular nature of the magnetism of these molecules [88].

Let us now closely examine the foundations of the single-ionic magnetism of LnPc_2_, using bis-phtalocyaninato terbium(III), TbPc_2_, as an example. The presence of the ligand field causes the Tb(III) ion to possess a split J = 6 ground multiplet with J${}_{z}$ = ±6 sublevels that are well isolated from excited states. The J${}_{z}$ = ±5 sublevels are more than 600 K above the ground state doublet. The uniaxial magnetic anisotropy is oriented perpendicularly to the phthalocyanine planes, resulting in an overall high energy barrier. An avoided level crossing between the Jz +/− 6 sublevels is found at zero field, while multiple-level anticrossing occurs between −0.05 and 0.05 T due to the hyperfine interaction with the I = 3/2 Tb nuclear spin, which opens several possible tunneling resonances. Further transitions between the J${}_{z}$ = ± 6 sublevels may occur at higher fields mediated by phonons (known as the direct relaxation process). The observed magnetization behavior of TbPc_2_ in bulk crystals presents the following characteristics: I a strong Ising-type anisotropy; II molecules that reverse their spin around zero-field via tunneling; and III phonon-mediated direct transitions at higher fields. Moreover, the hysteresis loop depends on the field sweep rate as the probability that a molecule reverses its magnetization at a certain level crossing is given by the Landau–Zener mechanism. It is observed that the magnetization reversal starts at more negative fields for the slower rates, and the hysteresis loop is broadened around zero-field for the fast rates [89,90].

To cut a long story short, lanthanide complexes exhibiting SMM behavior possess a considerably large axial magnetic anisotropy. This anisotropy arises from fundamentally distinct mechanisms from those responsible for the well-established SMMs of 3d metal clusters. In the case of 3d metal cluster SMMs, the magnetic interactions among high-spin 3d metal ions within a molecule cause easy-axis-type magnetic anisotropy, which is represented by the negative zero-field-splitting constant **D**. Conversely, for lanthanide single-ionic nanomagnets, such anisotropy is the result of the ligand field surrounding the lanthanide ion [90].

It is worth noticing that LnPc_2_ units, just like Cr_7_Ni mentioned previously, are often used as building blocks to obtain larger molecular moieties. By following the synthesis route reported independently by Fukuda and Wang, it was possible to obtain Ln-Pc triple-deckers, quadruple-deckers, or structures with even more levels, described by the Ln_*n*_Pc_*n+1*_ formula [91,92,93,94,95,96]. By creating such complex entities, one can introduce more than one kind of lanthanoid ion, i.e., Tb^3+^ and Dy^3+^, which results in peculiar magnetic and structural properties, for example, the dual magnetic relaxation process. Double and multiple-decker SMMs were utilized to engineer spintronic nano-devices [97,98,99].

A distinctive group of lanthanide-based molecular magnets is cluster fullerenes exhibiting considerable potential for surface evaporation and presenting exciting prospects in the field of nanotechnology [100,101]. Within these compounds, the magnetic characteristics stem from the inner cluster, while the protective carbon cage serves to protect the magnetic core from chemical interactions. As a result, this arrangement minimizes scattering and hybridization with the surface.

We have thus showcased the most notable and renowned groups of SMMs and summarized their properties in Table 1. To emphasize its significance to the field, both historical and technological, and to simultaneously draw an appropriate starting point for the depiction of molecular magnetism, we paid the most attention to the archetypal class of SMMs—Mn_12_. Then, we explored the next most prominent kinds of molecular nanomagnets, starting with star-like iron-based magnetic compounds, through heterometallic chromium-based rings, and ending with lanthanide single-ion magnets. It is now reasonable to move on to introducing nanostructured platforms for their deposition in the following section.

## 3. Types of Nanostructures as Substrates for SMMs

Since the type of substrate and method of SMM attachment plays a key role in the resulting nanocomposite, it is important for the following discussion to analyze the nanostructures that are commonly used in SMM deposition.

**Figure 4:**
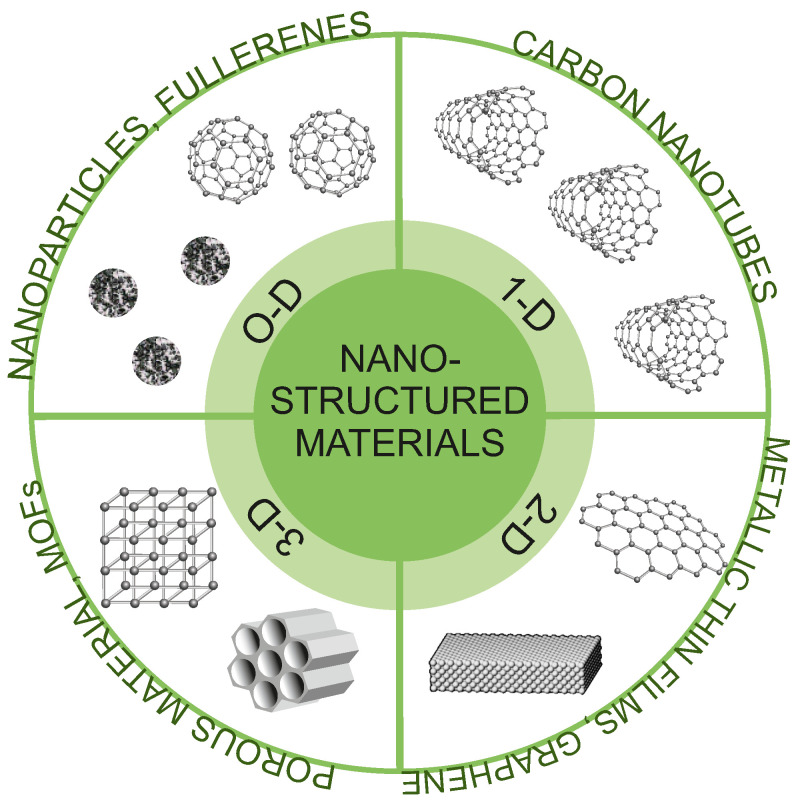
Types of nanostructured materials applied for SMMs deposition.

In general, nanostructures can be classified into different categories based on their composition, size, shape, and morphology. In this work, we have applied the classification proposed by Pokropivny et al. [108]. This classification includes four categories: 0D, 1D, 2D, and 3D (Figure 4). For 0D structures, all three dimensions are in the nanometric range. Among them, we can mention nanoparticles, well-dispersed nanopowders, or fullerenes. 1D structures are characterized by two dimensions on the nanoscale, while the third remains large. These types of structures have elongated shapes (e.g., nanotubes, nanoribbons, or nanobelts). 2D structures have only one dimension at the nanoscale. Among them, we can mention nanofoils, nanocoatings, or nanolayers. The last group of 3D structures includes structures in which all three dimensions are outside the nanoscale and nano dimensionality is only revealed in their internal structure. The group of these materials includes all porous structures (mezo- and microporous). It is obvious though that such classification is a conventional simplification as alleged 0D nanoparticles and fullerenes have their own 3D volume on a sufficiently small scale, as for nanotubes, nanobelts, and pores inside nanostructured silica.

There are a couple of strong arguments for the idea of nanostructures as suitable platforms for depositing SMMs. First, nanometric structures possess a high surface-to-volume ratio, providing a significantly larger surface area compared to bulk materials [109,110]. This feature allows higher-density nanomagnets to be deposited, increasing the chances of effectively measuring the system’s response to external stimuli. Moreover, it is possible to engineer and adjust the available surface by controlling the shape, size, and properties of given nanostructures during the synthesis stage [111,112,113]. Such control can enable the organized assembly of SMMs on the nanostructure surface, ensuring uniform the distribution and alignment of the molecules if prepared correctly [114]. Additionally, the interaction between SMMs and the surface of the nanostructure can create a protective barrier, especially when the process of encapsulation is involved, preventing the loss of magnetic properties and minimizing the disadvantageous influence of the external factors [12,109]. Finally, by selecting appropriate nanostructures, it is possible to modulate the interactions between the SMMs and the substrate or host system. Such interactions, emerging from electrical conductivity, and the magnetic or topological properties of the latter, can influence the magnetic properties, such as the magnetic anisotropy and relaxation dynamics of the molecules [110,115].

In analyzing possible nanostructured substrates for the deposition of single-molecule magnets, we will start with the ‘most nanometric’ ones, 0D structures, because their size is often comparable to the SMM being deposited, making the properties of the resulting nanocomposites potentially unique. Among them, it is reasonable to outline two groups—nanoparticles [116,117] and fullerenes [118,119]—because of their profusion in constituting hybrids with SMMs.

Nanoparticles may come in a variety of different shapes, sizes, and chemical compositions. They exhibit large specific surface area, ease of organization on surfaces, and reliable solubility depending on particles’ composition—features that make them promising candidates for functionalization with SMMs, which we will try to prove by relying on particular literature examples later on in the next sections. Within the field of molecular magnetism, the most frequently employed nanoparticles are those consisting of gold [24,120,121,122], iron or iron oxide [116], and silica [114,123].

The other group, fullerenes, are spherical carbon-based entities that exhibit exceptional mechanical and thermal properties and are excellent conductors of both electricity and heat [124]. Furthermore, fullerenes can dissolve in common solvents at room temperature (e.g., 1-chloronaphthalene [125,126,127]). The solubility of fullerenes is a useful feature when there is a need for their derivatization in order to allocate magnetic units or support fullerenes’ orientation and assembly [128,129]. Like nanoparticles, fullerenes have a high surface-to-volume ratio, making them an excellent material for use as substrates for the deposition (or encapsulation) of single-molecule magnets.

Increasing the dimensionality, we move to 1D materials. The most prolific examples of such structures used as substrates for SMMs deposition are nanotubes. In the field of molecular magnetism, carbon nanotubes have become the most popular [130]. They exhibit a variety of extraordinary thermal, physical, optical, and mechanical properties. Single-walled carbon nanotubes (SWCNT) exist, as well as multi-walled carbon nanotubes (MWCNT). While MWCNTs always conduct electricity and achieve at least the same level of conductivity as metals, the conductivity of SWCNTs depends on their chiral vector: they can behave like a metal and be electrically conductive; exhibit semiconductor properties; or be non-conductive [131]. They also exhibit excellent thermal conductivity and extreme tensile strength [132]. It is possible not only to anchor single-molecule magnets on carbon nanotubes but also to encapsulate them into such structures. In both cases, the functionalization of nanotubes is necessary, carried out in such a way as to avoid defects on the surface of the nanotubes and not change their sp^2^ electron structure and thus conductivity [133].

Increasing the dimensionality once again, we move to 2D structures. These are often referred to as thin films. One of the main advantages of thin layers is that they can be designed to exhibit unambiguous properties, such as electrical conductivity [134], optical reflectance [135], or mechanical strength [136]. Thin films constitute an excellent platform to accommodate SMMs and are being widely used for that purpose. One of the key advantages of thin layers for SMM deposition is their anisotropy, which enables them to control the orientation and arrangement of the molecules. When deposited on a thin film surface, magnetic molecules can form well-ordered arrays with precise spacing and orientation, which is important for applications such as magnetic memory devices and spintronics. Then, thin films have the ability to provide a stable and controlled environment for SMMs. The surface of a thin film can be engineered to provide specific chemical and physical properties, such as chemical reactivity, electronic structure, and magnetic anisotropy. This can be important for tuning the properties of the SMMs and controlling their interactions with the surrounding environment. Last but not least, thin films provide a platform for the study of the fundamental properties of SMMs. By controlling the thickness, composition, and structure of the substrate layer, researchers can investigate the effects of these factors on the magnetic properties of the SMMs. This can provide valuable insights into the behavior of SMMs at the nanoscale and help guide the design of future SMM-based devices.

The most popular example of a thin film is undoubtedly graphene. It is a single-atomic layer of graphite, ordered hexagonally, with carbon atoms being bonded with every three neighbors via firm σ-bonds. Charge carriers in graphene exhibit linear energy-momentum dependence, unlike the quadratic relationship observed in other materials, and graphene-based field-effect transistors can exhibit bipolar conduction. Charge transport in graphene is ballistic over long distances, and the material displays large quantum oscillations and strong, nonlinear diamagnetism [137]. In terms of conductivity, graphene is highly efficient in terms of both heat and electricity along its plane [138,139,140].

Graphene’s electronic properties make it particularly revolutionary as it has the potential to enable the development of faster and more efficient electronics [141,142,143,144]. Treating graphene as a thin film nanostructure, we can tell that it creates a great substrate material for magnetic molecules, which is especially useful for obtaining composite nanoelectronic or spintronic nanomaterials, due to its remarkable electrical features.

However, there are many other relevant examples of 2D materials with applications for immobilizing single-particle magnets beyond the scope of this paper. Among them, one can mention thin films of silicon [145,146], of silica [147], or the increasingly used yttrium-iron garnet (YIG) thin layers [148]. The latter structure is particularly important because it enables the communication between molecular magnets through spin-wave propagation [149,150]. This topic, however, is so vast that a separate study should be devoted to it.

In turn, 3D nanomaterials are excellent matrices for locating molecules with specific properties, including the SMMs discussed in this paper. These materials are characterized by a porous structure consisting of a system of pores with a well-defined narrow range of sizes connected by channels. Finally, the resulting material gains a huge specific surface area, providing space to accommodate a large number of single-molecule magnets with their simultaneous separation.

An important example of 3D materials can be metal–organic frameworks (MOFs). MOFs are a class of porous nanomaterials that consist of metal ions or clusters linked by organic ligands to form extended 3D structures with high surface area and tunable pore size. One of the most notable properties of MOFs is their high porosity and tunability, meaning their pore size, surface area, and chemical composition can be easily modified by changing the metal and organic ligands used in their synthesis [151].

The next class of 3D nanostructures, often employed as hosts for magnetic molecules, is mesoporous silica [152]. In particular, ordered mesoporous silicas play an important role as substrates for the spatial distribution of various molecules, including magnetic ones. First of all, mention should be made of the breakthrough material MCM-41, which has 2D ordered pores of about 2 nm in diameter, reaching 1000 m^2^/g of specific surface area [153,154]. A somewhat improved version of this material is SBA-15, which has wider pores (up to 6 nm) and improved stability [155,156]. Both of the materials exhibit the excellent accessibility of the inner volume of nanostructures through cylindrical pores so that they can be used as templates. Mesoporous silicas’ advantages are also their high thermal and hydrothermal stability and chemical inertness. It should also be noted that the ease of functionalization of silica with different anchor groups significantly facilitates their functionalization by SMMs.

At the end of this section, we should mention very specific nanomaterials, which are extremely helpful for immobilizing various molecules, including SMMs. These are structured thin films, whose channels can be spatially ordered. These types of materials have great advantages over powder materials. They allow not only for separation but also the spatial organization of deposited molecules, which seems to be a very important element in the development of nanoelectronics and spintronics. The most widely used material of this type is thin films of porous silica [157]. They can have a three-dimensional pore structure [158]; however, for sophisticated applications in nanoelectronics, the most interesting ones are those having 2D ordered pores [159,160], due to the possibility of ordering single-molecule magnets in the plane of the substrate, forming a nanometric arrangement of magnetic units. In this case, a thin silica film with appropriately ordered pores is a kind of template, on which the anchoring points for magnetic molecules are marked [161].

## 4. Deposition of Single-Molecule Magnets on Nanostructures

The attachment of SMM molecules to a substrate is one of the key steps in research conducted on nanomagnets. Therefore, a lot of work is focused on the selection of the synthesis method, the proper preparation of substrates, and the development of a methodology to confirm the success of the procedure.

**Figure 5:**
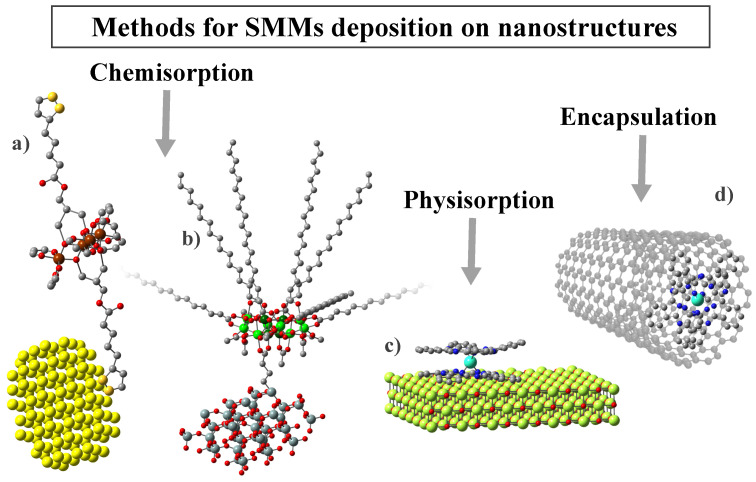
Examples of different methods for molecules attached to the surface: (**a**) chemisorption with an anchoring group of molecule (Fe${}_{4}$ SMM with organosulfur, H${}_{3}$thioctic ligands deposited on the Au), (**b**) chemisorption with anchoring group of substrate (Mn${}_{12}$-st molecule anchored to the silica with propyl-carbonic acid group), (**c**) physisorption of SMM to the surface (TbPc${}_{2}$ SMM adsorbed on MgO thin film), and (**d**) encapsulation into carbon nanostructure (TbPc${}_{2}$ SMM into SWCNT).

As presented in the previous section, one criterion for dividing nanostructures into categories is their dimensionality. Based on it, in Table 2, we have collected the most notable and described in this work examples of SMM deposition on substrates from all four categories (0D-3D). Nevertheless, the intricate characteristics of SMMs and their interactions with various nanostructures necessitates a more comprehensive examination of composites derived from them, along with an exploration of their properties. Therefore, further we will be focusing mainly on the nature of the substrate rather than its dimensionality. This approach will allow us to identify the most important features that characterize the given groups of substrates, as well as to indicate their influence on the resulting SMM/substrate complexes. Thus, for example, gold or carbon substrates enable current transport in the final composites. In the case of silica materials, the focus was on the magnetic and electrical inertness of these structures, which allows for greater confirmation of the efficiency of the synthesis carried out. On the other hand, the last group presented molecular self-organization in the form of single particles or monolayers on non-magnetic (magnesium oxide), or magnetic (iron oxide) substrates. In turn, the hybrids obtained with MOF structures show the presence of long-range and slow magnetic relaxation simultaneously. This makes it possible to study the interaction between magnetic matrices and SMMs in detail. Moreover, the deposition process varies depending on the substrate material and the specific SMM, leading to distinct interactions between the components (Figure 5). Molecules can be accommodated through either nonspecific van der Waals forces (known as physical adsorption) or specific chemical interactions that result in significant changes in electronic states (referred to as chemical adsorption). Additionally, interactions may involve noncovalent π–π stacking (supramolecular interactions) or encapsulation within the inner spaces of nanostructures.

### 4.1. Synthesis Methods and Main Characteristics

#### 4.1.1. Deposition on the Gold Substrates

With the development of SMM deposition, it has been observed that gold is one of the most attractive substrates, resulting in a high percentage of pioneering research in this field, conducted on both nanoparticles and thin films of gold [120].

The atomically smooth gold terraces are highly reactive, and magnetic molecules containing sulfur groups can be conveniently applied to these flat regions. Early reports in this direction were presented in 2003 by Cornia et al. when single-molecule magnets were deposited on a conducting substrate, Au(111) thin film, for the first time [162]. To achieve this, researchers took advantage of the strong Au-S bonding and introduced 16-(acetylthio)hexadecanoic acid, (L), groups to substitute acetate groups in Mn_12_, thus obtaining a highly soluble inorganic solvent [Mn_12_O_12_(L)_16_(H_2_O)_4_] derivative. Then, the deposition of nanoclusters on the Au(111) surface was carried out by combining a dilute solution of [Mn_12_O_12_(L)_16_(H_2_O)_4_] with tetrahydrofuran and NH_4_OH, which facilitated the deprotection of thiol groups and increased the chances of secure attachment to the gold surface (Au-thiolate bond). This method has demonstrated great efficacy in overcoming the oxidative vulnerability of organic thiols during the formation of self-assembled monolayers [163]. SQUID-supported AC susceptibility measurements revealed that during the ligand-exchange reaction, the Mn_12_ core remained unaltered as the energy barrier was found to be very similar to that of Mn_12_ before functionalization. Also, the temperature dependence of the spin relaxation time was measured, and the results indicated that magnetization reversal likely occurs through a thermally activated mechanism. A similar strategy using thiol groups was used to obtain Mn_12_-cysteine-Au NPs nanocomposite material [24]. The strong attraction between gold and sulfur can be realized by using Au nanoparticles with a hexadecylamine (HDA-A) shell. Additionally, HDA provides compatibility with SMM and suitability for exchange reactions [164]. An example is the work of Cornia and co-workers, who grafted Fe_4_ particles onto Au nanoparticles (∼5 nm in diameter) [120], as schematically shown in the Figure 5a. For depositing Fe_4_-thioctic, derivatives were used, having a propeller-like structure thanks to two tripodal ligands obtained by the esterification of (±)-α-lipoic acid (‘thioctic acid’) with pentaerythritol. Next, the Fe_4_-NPs hybrids were constructed through the replacement of HDA ligands with Fe_4_-thioctic in a solution of CH_2_Cl_2_. The magnetic bistability of the final product was confirmed using the X-ray magnetic circular dichroism (XMCD) technique.

The anchoring of S-ligand groups on gold is also justified for the formation of complexes with lanthanides and chromium-based rings. Such an effect was obtained in the case of the deposed Dy_2_(Hhmb)_3_(NCS)_3_]·2MeOH·Py, which has retained its magnetic properties while exhibiting a change in AC magnetic characteristics, especially in QTM [122]. In turn, the behavior of slow relaxation and the appearance of butterfly-like hysteresis were observed in the case of TbPc_2_ [121]. Corradini et al., using liquid deposition, successfully anchored the Cr_7_Ni rings on the surface of Au(111) while maintaining magnetic properties. In their study, they have prepared different Cr_7_Ni derivatives—those containing sulfur (i.e., Cr_7_Ni-thio: [HSC_2_H_4_NH_3_][Cr_7_NiF_8_(O_2_CtBu)_16_] and Cr_7_Ni-4mtpp: ([(Et)_2_NH_2_][Cr_7_NiF_8_(O_2_CCH_2_tBu)_15_(O_2_CCH_2_CH_2_C_6_H_4_SCH_3_)]) and those free of sulfur (Cr_7_Ni-pyridine [PyCH_2_NH_2_Et][Cr_7_NiF_8_(O_2_CtBu)_16_]). What they have proven is that incorporating S-containing ligand groups for anchoring on gold is also an appropriate approach for Cr_7_Ni nanomagnets as the obtained percentage of the covered Au area (determined by means of STM—scanning tunneling microscopy) grows from roughly 2% in the case of Cr_7_Ni-pyridine to about 25–35% in the case of Cr_7_Ni-4mtpp [165].

#### 4.1.2. Deposition on the Carbon Substrates

Carbon materials, used as nanostructured substrates in the field of molecular magnetism, are very widely reported in the literature. In this review article, we divide them in terms of three categories based on their dimensionality (0D, 1D, and 2D). We decided to start the review of this part with 1D carbon nanotubes. These materials, due to their structure, can be characterized by different conductive or mechanical properties, which is an extremely attractive feature from the point of view of conducting research on magnetic properties.

The process of depositing SMMs on the surface of nanotubes is carried out after their prior functionalization, which can contribute to a number of changes in their structural, chemical, or mechanical properties. Particular attention is paid to possibly formed distortions, loss of symmetry, and changes in the electron structure of sp^2^. Coronado et al., in their paper, proposed two possible chemical ways, one-step and two-step, for the electrostatic grafting of cationic Mn_4_ ([Mn_4_(O_2_CCH_3_)_2_(pdmH)_6_]^4+^, pdmH—deprotonated pyridine-2,6-dimethanol [166]) onto the surface of functionalized, anionic MWCNTs [167]. The first, two-step route began with introducing carboxylic groups to the outer walls of the MWCNTs. Then, these groups underwent deprotonation and were further linked to the cationic Mn_4_ complexes. The second approach was a direct attachment. The cationic, magnetic complexes were combined with in-situ-generated anionic MWCNTs. Carboxylic groups acted as defects on the nanotubes’ surface and altered their sp^2^ electronic structure, so the hybrids produced via the first route were conducting poorly. In contrast, the second approach resulted in the enhanced electrical conductivity of the hybrid relative to the pristine MWCNTs as it involved the n-type doping of the nanotubes. Experimental techniques utilized in the study revealed that the second route yielded a higher degree of grafting compared to the first route, and the grafting process, although the superparamagnetic behavior of the Mn_4_ was retained, noticeably affected the magnetic response, probably due to surface effects—distortion and loss of symmetry. In their 2013 article, Ganzhorn and coworkers described a noninvasive grafting technique of an SMM onto a carbon nanotube NEMS (nanoelectromechanical system) [168,169], which preserved both the mechanical properties of the carbon nanotube NEMS and the magnetic properties of the SMM [170]. They demonstrated that the nonlinearity of a carbon nanotube’s mechanical motion could be utilized to investigate the reversal of a molecular spin, giving experimental evidence for detecting a single spin through a mechanical degree of freedom at the molecular level, thus showing that CNTs may be used as magnetometers for SMMs.

J. Galan-Mascaros, et al. published a paper summarizing the successful incorporation of ‘double-deckers’ TbPc_2_ into single-walled carbon nanotubes (SWCNTs) [171]. π-π interactions were employed, and one of the Pc ligands was substituted for the pyrenyl group as a linker strongly interacting with SWCNTs. Anchoring was realized by dispersing SWCNTs in a solution of a heteroleptic, pyrene version of TbPc in saturated CH_2_Cl_2_. As it turned out after magnetic characterization, the superparamagnetic behavior of molecular magnets in the conjugates was retained. Moreover, the AC susceptibility measurement of composites showed that the imaginary part of magnetic susceptibility (χ”) arises in even higher temperatures as for a bulk sample of TbPc_2_, with maxima between 44 K (997.3 Hz) and 27.5 K (1.00 Hz). Another report on TbPc_2_ on CNTs from 2017 revealed that such composite systems exhibit a giant magnetoresistance phenomenon [172].

In turn, Bogani et al., for the anchoring on nanotubes, used pyrene-functionalized Fe_4_ molecules with dipivaloylmethane ligands [66]. The pre-synthesized CNTs and SMMs were connected together by the immersion of CNTs in a solution of Fe_4_ in 1,2-dichloroethane (DCE). In their study, they achieved precise control over the grafting of SMMs at the individual molecule level, showcasing the remarkable sensitivity of CNT-FETs (field-effect transistors) to single SMMs. These findings laid the foundation for the development of ‘double-dot’ molecular spintronic devices [173], where a specified quantity of nanomagnets is connected to an electronic nanodevice. Additionally, they enabled the exploration of the magnetic Coulomb effect. The other paper released that year was published by Giusti et al., and it presented the magnetic bistability of Fe_6_-POM (Na_6_((CH_3_)_4_N)_4_[Fe_4_ (H_2_O)_2_(FeW_9_O_34_)_2_]·45H_2_O, POM—polyoxometalate [174]) exhibited by these complexes after grafting them onto the SWCNTs’ surface [175]. The attachment of POM molecules was easily accomplished through the use of sonification on a suspension of nanotubes in a buffer of dichloroacetic acid that included the Fe_6_-POM. They thus demonstrated that the noncovalent grafting of an SMM complex on SWCNTs does not alter the integrity of the molecules, as evidenced by various complementary techniques. The compelling outcome was that the individual molecules exhibited a slow relaxation of magnetization, resulting in magnetic bistability at the level of single molecules.

It is possible not only to anchor single-molecule magnets on carbon nanotubes but also to encapsulate them into such structures. This type of molecular architecture makes the carbon cage protect the magnetic elements (objects) from decoherence by environment noise and allows for the stabilization of magnetic bonds and interactions. For example, recently TbPc_2_ nanomagnets have been successfully encapsulated for the first time into SWCNTs, as the 2021 study reports [176] (see Figure 5d). The encapsulation was carried out using a capillary method. Although the assumed one-dimensional chain stacking of magnetic molecules inside nanotubes was not confirmed by means of the experimental methods used in the study, the SMM behavior of TbPc_2_ was not destroyed during the encapsulation and the final TbPc_2_@SWCNT product retained magnetic properties. Also, Mn_12_ molecules can be encapsulated into carbon nanotubes, as the study from 2011 shows [177]. As host systems, the authors chose graphitized multi-walled carbon nanotubes (GMWCNT), generated through catalytic chemical vapor deposition. To enable the encapsulation of Mn_12_-ac SMM, they pre-treated the GMWCNT with concentrated nitric acid, followed by heating in air. This process yielded GMWCNTs opened on one side. For the transport of SMM molecules into the nanotubes, they utilized supercritical CO_2_ (scCO_2_). The resulting hybrid material, Mn_12_-ac@GMWCNT, underwent a series of structural and magnetic measurements, which confirmed the proper incorporation of SMMs into GMWCNTs, showed its magnetoresistance, and proved that the SMM behavior was retained.

In the case of 2D graphene sheets, like for CNTs, it is possible to modify their surface by anchoring appropriate functional groups (ligands) on their surface. Such an approach can affect the conductive properties of the substrate, making it possible to anchor SMMs, as a consequence of which it is possible to carry out magnetic measurements of the obtained complexes. In 2010, Lopes et al. were the first to report on the assembly of lanthanide-based nanostructures on graphene [178]. The authors, bearing in mind the maintenance of the properties of both components, decided to employ π-stacking interactions as a binding force. To achieve this, prior to anchoring, one of the phthalocyanine macrocycles in each TbPc_2_ complex was exchanged for one pyrene and six hexyl groups as both of these molecules are known for demonstrating a favorable interaction with sp^2^ carbon materials, optimizing the intermolecular van der Waals forces [179]. SIMs prepared in such a manner were introduced onto the graphene flakes by drop-casting the TbPc_2_ solution in dichloromethane. Owing to Raman spectroscopy and atomic force microscopy, a weak electronic interaction between graphene and TbPc_2_ molecules was observed. Only a minor charge transfer occurred, causing a Fermi-level shift while maintaining the mobility of graphene. The experimental results were supported by DFT calculations, which indicated van der Waals coupling between pyrene and graphene. This suggested that the electronic properties of TbPc_2_ and graphene remained largely unaffected, even at low molecule densities where no molecular clusters were detected.

Zhu et al. swapped R = CH_3_ groups for R = CHCl_2_ in the [Mn_12_O_12_(O_2_CR)_16_(H_2_O)_4_] and deposited both species on CVD-grown graphene. By means of gate-voltage-dependent magnetotransport measurements, they showed that R = CHCl_2_ ligands, which are more electronegative, remarkably change the electronic transport in graphene and thus its conductive properties, while the original, acetate ligands only minimally alter the conductivity of graphene [180]. A couple of years earlier, in 2014, it was shown by means of theoretical DFT (Density Functional Theory) calculations that besides R = CH_3_ and R = CHCl_2_, it is possible to functionalize graphene with Mn_12_-C_6_H_5_ and Mn_12_-H derivatives [181].

In this work, the hybrids were generated through the non-covalent attachment of [Fe_4_(L)_2_(dpm)_6_] (Hdpm = dipivaloylmethane and H_3_L = 2-hydroxymethyl-2-(4-(pyren-1-yl)-butoxy)methylpropane-1,3-diol) to exfoliated graphene sheets in the presence of two pyrene groups. The study has shown that substrate effects on quantum dynamics can serve as a methodological tool to study spin-substrate interactions and reveal symmetry-breaking quantum effects.

Gragnaniello et al., using the electrospray method, showed that Fe_4_H can form highly periodic, self-assembled arrays on graphene/Ir(111) substrates [182]. The results of XMCD measurements have shown that the magnetic easy axis exhibits uniaxial out-of-plane orientation, and the magnetic anisotropy of the deposited molecules matches that of the bulk material. Thus, the Fe_4_H complex maintained its bistable magnetic behavior within the 2D superlattice on the graphene surface, despite observed interactions with the surrounding environment. Recently, Paschke and coworkers further investigated the Fe_4_H-graphene/Ir(111) system with the use of inelastic electron tunneling spectroscopy [183], to address individual and collective Fe_4_ SMM magnetic properties, like the exchange interaction constant between the iron atoms, determined to be J = 1.5 ± 0.4 meV [184].

The last group of carbon substrates presented in this review are 0D fullerenes. In the case of single-molecule magnets, the most commonly used are endohedral fullerenes, in which additional atoms, molecules, or ions are introduced inside the fullerene, for example, two dysprosium-containing mixed dimetallic sulfide clusterfullerenes, namely, DyScS@C_82_ (I, II) [185]. The authors of this paper, after synthesizing and characterizing the material, found that it would perform very well as an SMM. The isomers of DyScS@C_82_ exhibit very similar single-molecule magnetic behavior to open hysteresis loops at low temperature. The magnetic blocking temperatures are about 7.3 K, which is one of the highest recorded values for SMM clusterfullerene. Other examples of single-molecule magnets, presented by Junghans et al., are Dy_2_TiC@C_80_-(I,II) and Dy_2_TiC_2_@C_80_-I [186]. The authors showed that the presence of a second carbon atom in the Dy_2_TiC_2_@C_80_ cluster leads to significantly worse SMM properties. In 2021, it was reported by Paschke et al. that dimetallofullerene Dy_2_@C_80_(CH_2_Ph) endohedral complexes exhibit outstandingly high blocking temperature, 17 K, keeping the magnetization stable for 100 s [129]. The samples were arranged into islands on the graphene/Ir(111) surface with the help of the electrospray deposition, and their magnetic and electronic properties were examined using XAS and XMCD techniques.

SMMs can also be successfully grafted onto fullerene surfaces. An example is the archetypal Mn_12_, which has been successfully grafted by cocrystallization onto C_60_ fullerenes [187]. The study revealed that incorporating fullerene molecules into familiar compounds resulted in remarkable alterations in magnetization dynamics. These changes included the emergence of the ‘magnetization training’ effect and the subsequent displacement of the asymmetrical hysteresis loop along the magnetization axis while still preserving the properties of SMMs. These effects can be attributed to the orientational dependence of magnetism in organic substances doped with C_60_. Spree et al. demonstrated that by functionalizing Tb_2_@C_80_(CH_2_Ph) with a linker molecule terminated with pyrene, they were able to promote the formation of self-assembled monolayers (SAMs) on substrates such as graphene and highly oriented pyrolytic graphite (HOPG) [128]. These molecular layers displayed magnetic hysteresis up to 28 K, indicating that the functionalization or deposition process did not negatively affect the magnetic properties.

#### 4.1.3. Deposition on the Silica Substrates

While efforts to deposit SMMs on nanostructured gold or carbon-based nanostructures as the conducting substrates were made bearing in mind current-transport features of the final composite nanomaterials, different approaches took advantage of the electric and magnetic inertness of other host systems, such as silica, SiO_2_, in various forms. The literature examples show that two main types of nanostructured silica, namely, 3D mesoporous silica matrices, and 0D silica nanoparticles were most commonly applied for the SMM deposition. Probably the first attempt to incorporate magnetic clusters into porous silicates was made by T. Coradin et al. in 2002, who proposed this type of nanocomposite as a first step in the development of high-density memory storage materials. They used mesoporous SBA-15 type silica [188] as a host for polynuclear complexes belonging to the families of Mn_12_ and Cr-carboxylates. Their results showed no relevant difference in the insertion rate between the non-functionalized matrix and the functionalized matrix with ethylenediaminetriactic acid; however, in both cases the signature of single-molecule magnet observed for bulk Mn_12_ or Cr-carboxylates is still present in the nanocomposite [189]. One year later, the same group analyzed molecular clusters based on 12 manganese ions exhibiting single-molecule magnet behavior incorporated inside SBA-15-type silica. The authors considered the possibility of introducing magnetic complexes into a silica matrix from a geometrical point of view, concluding that the largest of the derivatives with a diameter of about 17 Å Mn_12_O_12_(CH_3_COO)_16_(H_2_O)_4_]·2CH_3_COOH·4H_2_O could not be introduced into a structure containing pores with a diameter of 25 Å, while the maximum filling of the pores was observed for silica with pore diameters around 60 Å. As in previous reports, the magnetic properties of the magnetic clusters were retained upon incorporation into the silica matrix [190]. In the same year, 2003, M. Clemente-Leon et al. studied the incorporation of four Mn_12_ derivatives into the channels of the MCM-41 silica [191,192]. The authors pointed out that using silica matrices would enable obtaining 1D ordered arrays of these magnetic clusters within the hexagonal channels of the mesoporous silica. Such organization of Mn_12_ clusters in one-, two-, or three dimensions is a crucial step in the search for applications. Their research showed that only the smallest clusters exhibiting compatible size with the pores of MCM-41 could enter into the mesoporous silica. Moreover, the magnetic properties of Mn_12_ clusters remained intact after MCM-41 impregnation. Laskowska et al. combined SBA-15 with Mn_12_-stearate—a derivative much more soluble in organic solvents and resistant against water-catalyzed reduction than its original Mn_12_ counterpart [50]. Via the prefunctionalization of SBA-15 with propyl-carboxyl anchoring groups, it was possible to precisely cover the channels of silica with molecular magnetics and control their concentration and distribution. Magnetic measurements showed a gradual reduction in magnetic performance with concentration of deposited SMMs.

Also, other types of mesoporous silica, namely, bimodal UVM-7, nanoparticle xerogel UVM-11, and the aforementioned MCM-41, have been adopted as carriers for molecular nanomagnets. In their work [193], Pardo et al., as guest magnetic molecules, used the Ni_8_ octanuclear nickel(II) oxamate complexes with the full chemical formula [Ni_2_(mpba)_3_][Ni(dpt)(H_2_O)]_6_(ClO_4_)4·12.5H_2_O, where mpba—m-phenylenedioxamate (a bridging ligand) and dpt—dipropylenetriamine (a terminal ligand). The preparation of this complex involved a systematic approach known as the ‘complex-as-ligand’ strategy, enabling the deliberate construction of metal coordination cages. This method utilizes self-assembled, exchange-coupled metallacyclic complexes with aromatic oligooxamate ligands as building blocks, allowing for the rational design of the desired complex [194,195]. Host materials were synthesized using the ‘atrane route’, in which silicon atrane complexes (silatrane) were employed as hydrolytic inorganic precursors and surfactants as porogen species [196]. All three composite materials were prepared by a simple, one-pot method of dissolving Ni_8_ in water and adding an appropriate amount of MCM-41/UVM-7/UVM-11. Magnetic measurements conducted on the final composite product showed that not only did it exhibit slow relaxation of magnetization in higher blocking temperatures but also exhibited extraordinary spin-glass behavior.

Another report on the deposition of single-molecule magnets on the nanostructured silica, namely, silica nanoparticles, was published by Allouche et al. The lanthanide-based SMMs of Dy(III) were deposited on silica nanoparticles using a simple and scalable two-step process and showed magnetic hysteresis at liquid ${}^{4}$He temperature [123]. The synthesis process relies on the first grafting of Dy(III)-based SMMs on the partially dehydroxylated silica and then the thermal annealing of material under high vacuum for the removal of organic ligands and formation of isolated Dy(III) ions. Silica nanoparticles were also used as a substrate for the deposition of Mn${}_{12}$ SMMs (see Figure 5b). In this case, magnetic molecules of Mn${}_{12}$-stearate were connected to the Si NPs, prepared according to the Stöber protocol [197], by means of wet chemistry [114]. Propyl-carbonic acid links were applied as anchoring units, while trimethylsilane groups allowed one to control the distribution of SMMs. Long, stearic acid chains as ligands for the molecules’ magnetic cores facilitated the separation of nanomagnets on the surface, helping to avoid the formation of agglomerates, and SQUID magnetometry confirmed that the final product retains the magnetic properties of the initial manganese clusters [115].

#### 4.1.4. Other Substrates for SMMs Deposition

The examples presented above indicate that gold, carbon, and silica nanostructures appear to be the most suitable substrates for molecular magnet deposition, due to the ease of synthesis and potential applicatory properties of final nanocomposites. Nevertheless, recent developments in approaches and chemical strategies have opened the door to considering alternative substrate types for immobilizing SMMs, each with its unique advantages.

The self-organized arrays of Fe${}_{4}$ SMMs were successfully formed on the thin layer of hexagonal boron nitride (*h*-BN) on Rh(111) by applying the electrospray deposition method [18]. To achieve the flat adsorption geometry of molecules, the authors synthesized Fe${}_{4}$-derivative with the smallest tripodal ligand possible (Fe${}_{4}$H). The authors indicate that the method used makes it possible to obtain molecular self-assembly in the form of single isolated molecules, small islands, or even an almost perfect monolayer. Another study reports on the adsorption of TbPc${}_{2}$ SMMs on the nonmagnetic, insulating MgO thin film [198] (see Figure 5c). Similarly to the previous study, attached molecules form uniform ensembles by self-assembling on the surface with out-of-plane easy axes. Recent investigations show the possibility of depositing SMMs also on magnetic substrates like iron oxide NPs [116]. Here, heterometallic 3d–4f SMM [Co_4_Dy(OH)_2_(SALOH)_5_(chp)_4_(MeCN)(H_2_O)_2_], Co-Dy, was immobilized onto iron oxide NPs using an oleate self-assembled monolayer as a surfactant. Interestingly, although pristine NPs and SMMs are both magnetic, the composite product exhibits a magnetic hysteresis that cannot be described as the simple sum of hysteresis curves of Co-Dy and FeO NPs measured at the same temperature.

The metallic thin film surface (e.g., gold, silver), often additionally coated with a thin inorganic film, aims to optimize the interaction between single-molecule magnets and the substrate [199,200]. Such a decoupling layer can minimize the hybridization of the metallic substrate, which is responsible for quenching the typical magnetic bistability. Sorrentino et al. deposited terbium(III) bis-phthalocyaninato (TbPc${}_{2}$) complex on an Ag(100) substrate, on the surface of which a TiO${}_{2}$ monolayer with a lepidocrocitelike structure was grown [201]. By employing X-ray magnetic circular dichroism, it was demonstrated that the TiO${}_{2}$ film successfully retains the molecular spin character. Additionally, magnetic measurements revealed the presence of quantum magnetization tunneling in the SMM/substrate complex. The most recent study report absorption of the TbPc${}_{2}$ single molecule was on the Cu(100) surface covered with NaCl thin film [202]. The authors pointed out that two-monolayer NaCl decouples the electronic interaction from the metal substrate suppressing the charge transfer in ESR-STM measurements. Such a method has great potential for the detection and manipulation of the hyperfine-coupled Tb spins in the TbPc${}_{2}$ molecule, minimizing decoherence due to the coupling to the metal substrate.

Lately, the use of MOF structures for the deposition of SMMs has been of special interest. The first attempt to incorporate SMMs into MOFs’ pores was made in 2015, by Aulakh et al. [203]. The magnetic molecules chosen were archetypal Mn_12_-ac, and the metal–organic framework used was aluminum-based [Al(OH)(SDC)]_n_ (H_2_SDC = 4,4’-stilbenedicarboxylic acid), CYCU-3 for short [204]. This particular metal–organic framework has good solvent and thermal stability and contains hexagonal pores with a diameter of 3 nm, which is suitable for facilitating Mn_12_-ac molecules, which are approximately 1.6 nm in diameter. Moreover, another advantage is that the presence of diamagnetic aluminum centers does not impact the overall SMM properties of Mn_12_-ac. Structural studies performed on the final composite material revealed that there was no crystallization of magnetic molecules inside pores or on the surface of the MOF, but each pore included exactly one manganese molecule within a well-organized crystalline matrix, although not achieving complete loading. Furthermore, the magnetic analysis showed that the single-molecule magnetic behavior of the composite SMM@MOF material was completely retained and the thermal stability was substantially improved. Two years later, the same scientific group led by Aulakh went one step further and tested the possibility of placing seven different Mn_12_ derivatives in the CYCU-3 pores [205]. The ligand groups of these derivatives were: CF_3_, (CH_3_)CCH_2_, CH_2_Cl, CH_2_Br, CHCl_2_, CH_2_Bu^t^, and C_6_H_5_. The molecular dimensions of the molecules ranged from 1.8 to 2.2 nm, so even after ligand-exchange reactions all of them were still suitable for incorporation into frameworks’ pores. Successfully, after appropriate structural and magnetic measurements, every Mn_12_ derivative turned out to be correctly placed inside the pores (that is, without agglomerates or crystalline phases) and to possess slow relaxation of magnetization, indicating retained SMM behavior. Also, the main goal of the study was achieved—to demonstrate that the utilization of MOFs as platforms for nanostructuration of SMMs was not limited to a specific host–guest system but had the potential to be applied to numerous other molecular magnets. Recently, Aulakh took another MOF—so-called NU-1000 ([Zr_6_(μ_3_-OH)_8_(OH)_8_(tbapy)_2_] (tbapy = 3,6,8-tetrakis(p-benzoic acid)pyrene))—and successfully placed Mn_12_-ac in its pores. With the help of HRTEM, it was possible to directly image single magnetic molecules locked inside [206]. The obtained images provide direct and unequivocal evidence, for the first time, supporting the adsorption of molecular guests within the porous host.

There was also a report on the incorporation of yet another kind of molecular nanomagnet into an MOF, thus proposing a synthetic approach capable of integrating well-arranged SIMs into the pores of a magnetic MOF using a single-crystal to single-crystal (SC to SC) post-synthetic procedure [207,208,209]. By employing this approach, it became feasible to combine the long-range magnetic ordering of the magnetic MOF with the slow magnetic relaxation exhibited by the incorporated SIM. This fusion led to the formation of a unified solid, potentially resulting in an interplay between both magnetic phenomena. Molecular nanomagnets used in this work were Mn^III^-porphyrin ([Mn^III^(TPP)(H_2_O)_2_]ClO_4_ (TPP = 5,10,15,20-tetraphenylporphyrin)), and the metal–organic frameworks were chosen to be manganese(II)–copper(II) Na_4_Mn_4_[Cu_2_(Me_3_mpba)_2_]_3_·60H_2_O, where [Me_3_mpba^4−^ = N,N’-2,4,6-trimethyl-1,3-phenylenebis(oxamate)]. The novel, composite nanomaterial produced was [Mn^III^(TPP)]Na_3_Mn_4_[Cu_2_(Me_3_mpba)_2_]_3_·39H_2_O. This hybrid was synthesized through a straightforward diffusion and reversible cation exchange process in the solid state. This involved immersing crystals of MOF in a saturated water/methanol (5:1) solution of [Mn^III^(TPP)(H_2_O)_2_]ClO_4_. The final product was characterized structurally and magnetically. The obtained hybrid material, which showcases the simultaneous presence of a long-range magnetic order and a slow magnetic relaxation, offers an exceptional opportunity to conduct a thorough investigation into the interplay between the magnetic host matrix and the SIM guest.

### 4.2. Applications and Advantages

The above examples show that the deposition of single-molecule magnets onto surfaces frequently necessitates the accurate selection of the substrate, refinement, and modification of the molecule’s outer ligand structure, as well as the improvement of synthesis techniques. Attachment and separation can cause the distortion or decomposition of delicate magnetic molecules, leading to strong alternation or even loss of their magnetic characteristics during the deposition process [210,211]. However, nanostructured substrates provide a great means for the formation of nanocomposite materials with SMMs, offering a protective barrier for the preservation of their magnetic properties. Most of the considered examples confirm that both the static and dynamic magnetic behavior of molecules remains intact or just slightly modified after the deposition process (see Table 2). Moreover, there are reports showing the increased magnetic performance of SMMs after organization on specific substrates. A significant increase in coercivity in comparison to the bulk compound was reported for the TbPc${}_{2}$ molecules absorbed on the MgO thin film, which is probably explained by the suppression of QTM [198]. The appearance of butterfly-type hysteresis of the same molecules after deposition on the Au nanoparticles was also reported [121]. Similar hysteresis behavior was observed for the Dy(III) [123] and Mn${}_{12}$[114] SMMs deposited on the SiO${}_{2}$ nanoparticles, as well as for the Tb(III) [176] and Mn${}_{12}$ molecules encapsulated in carbon nanotubes [177]. These observations are most probably related to the separation and isolation of molecules on the surface and the decrease in intermolecular interactions. Let us note that a significant impact on the magnetic parameters improvement of SMMs was observed after their deposition on other carbon nanostructures. The following modifications in SMMs/nanostructure composites were reported: exceptionally high blocking temperature (around 7 K or 17 K for encapsulated Dy SMMs inside the fullerene cages [129,185] or even 28 K for fullerene/Tb SMMs monolayers on graphene [128]), an increase in the magnetic hysteresis (as a result of ‘magnetization training’ effect in the Mn${}_{12}$/fullerene complex [187]), the enhancement of anisotropy barrier and relaxation times (for Tb SMMs on SWCNT [171]), and the giant magnetoresistance effect (observed in carbon nanotubes with side-attached, terbium-based SMMs [172]).

**Table 2:** Literature examples of SMMs deposited onto nanostructures. We denote **H_c_** and **Δ/k_B_** as coercive field value and the magnetization reversal energy barrier, respectively.

Substrate Dimensionality	Substrate	SMM	Magnetism of the Composite	Literature
0D	Au nanoparticles	Mn${}_{12}$	H_c_ nor Δ/k_B_ not reported	[24]
		Fe${}_{4}$	H_c_ value not reported Δ/k_B_ = 8.0 K at H = 0.0 Oe Δ/k_B_ = 11.6 K at H = 1.0 kOe	[120]
		TbPc${}_{2}$	H_c_ = 0.0 Oe at T = 1.8 K Δ/k_B_ = 678.8 K	[121]
		[Dy${}_{2}$(Hhmb)${}_{3}$ (NCS)${}_{3}$]· 2MeOH·Py	H_c_ nor Δ/k_B_ not reported	[122]
	Fe${}_{2}$O${}_{3}$ nanoparticles	Co-Dy	H_c_ = 1.1 kOe at 2.0 K Δ/k_B_ value not reported	[116]
	SiO${}_{2}$ nanoparticles	Mn${}_{12}$	H_c_ = 1.0 kOe at 2.0 K Δ/k_B_ = 51.0 K	[114,212]
		Dy(III)	H_c_ value not reported Δ/k_B_ = 65.0 K at H = 0.0 Oe	[123]
	C${}_{82}$	DyScS	H_c_ = 6.0 kOe at T = 2.0 K Δ/k_B_ = 69.8 K at H = 0.0 Oe	[185]
	C${}_{80}$	Dy${}_{2}$TiC/ Dy${}_{2}$TiC${}_{2}$	H_c_ nor Δ/k_B_ not reported	[186]
		Dy${}_{2}$	H_c_ = 12.0 kOe at T = 2.5 K Δ/k_B_ = 615.0 K at H = 4.0 kOe	[129]
		Tb${}_{2}$	H_c_ = 54.0 kOe at T = 20.0 K Δ/k_B_ = 725.0 K at H = 70.0 kOe	[128]
	C${}_{60}$	Mn${}_{12}$	H_c_ = 2.5 kOe at T = 1.75 K Δ/k_B_ value not reported	[187]
1D	SWCNTs	TbPc${}_{2}$	H_c_ value not reported Δ/k_B_ = 504.8 K	[171]
			H_c_ nor Δ/k_B_ not reported	[172]
			H_c_ value not reported Δ/k_B_ = 590.0 K at H = 0.0 Oe	[176]
		Fe${}_{4}$	Δ/k_B_ nor H_c_ not reported	[66]
		Fe${}_{6}$-POM	H_c_ = 0.7/0.9 kOe at 0.5/0.04 K Δ/k_B_ value not reported	[175]
	MWCNTs	Mn${}_{4}$	H_c_ nor Δ/k_B_ not reported	[167]
		Mn${}_{12}$	H_c_ = 2.0 kOe at T = 1.8 K Δ/k_B_ = 57.0/30.0 K for slow/fast relaxing species	[177]
2D	Au(111) thin film	Mn${}_{12}$	H_c_ value not reported Δ/k_B_ = 55.1 K	[162]
	MgO thin film	TbPc${}_{2}$	H_c_ = 30.0 kOe at T = 3.0 K Δ/k_B_ not reported	[198]
	TiO${}_{2}$/Ag(100) thin film	TbPc${}_{2}$	H_c_ not reported Δ/k_B_ not reported	[201]
	NaCl/Cu(100) thin film	TbPc${}_{2}$	H_c_ not reported Δ/k_B_ not reported	[202]
	Graphene	Mn${}_{12}$	Not reported	[180]
		Fe${}_{4}$	H_c_ value not reported Δ/k_B_ = 13.8 K	[213]
		TbPc${}_{2}$	Not reported	[178]
	Graphene/ Ir(111)	Fe${}_{4}$	H_c_ = 0.0 Oe at T = 3.0 K Δ/k_B_ value not reported	[182]
	h-BN/Rh(111) thin film	Fe${}_{4}$	H_c_ = 0.0 Oe at T = 1.8 K Δ/k_B_ value not reported	[18]
	Si/W thin films	Cr${}_{8}$F${}_{8}$(piv)${}_{16}$	Not reported	[22]
3D	SBA-15 silica	Mn${}_{12}$	H_c_ = 4.0 kOe at T = 2.0 K Δ/k_B_ = 63.0 K	[189]
			H_c_ value not reported Δ/k_B_ = 70.0 K	[214]
		Mn${}_{12}$/ Mn${}_{4}$	H_c_ = 6.0 kOe at T = 1.8 K Δ/k_B_ = 72.0 K	[190]
		Mn${}_{12}$	H_c_ = 1.6 kOe at T = 2.0 K Δ/k_B_ = 55.2 K	[50]
	MCM-41 silica	Mn${}_{12}$	H_c_ = 1.5 kOe at T = 2.0 K Δ/k_B_ = 58.4 K	[192]
	MCM-41/ UVM-7/ UVM-11 silicas	Ni${}_{8}$	H_c_ = 0.8/2.99/0.24 kOe at T = 2.0 K Δ/k_B_ = 621.0/742.0/171.0 K	[193]
	CYCU-3 MOF	Mn${}_{12}$	H_c_ = 2.0 kOe at T = 1.8 K Δ/k_B_ = 57.0 K	[203]
	NU-1000 MOF	Mn${}_{12}$	H_c_ = 8.0 kOe at T = 1.8 K Δ/k_B_ = 69.1 K	[206]
	Mn(II)-Cu(II) MOF	MnTPP	H_c_ = 0.8 kOe at T = 2.0 K Δ/k_B_ = 14.4 K	[207]

The above observations validate the potential of nanostructures to serve as an excellent platform for hosting magnetic molecules, creating nanocomposite structures with the desired performance and significant practical applications. However, the ability to not only deposit molecules on the surface but also to control their distribution is another factor that must be addressed on the path to SMM practical applications. The ability to modify and access any individual molecule on the surface is required by modern nanoelectronics devices. However, only a few examples of nanostructures decorated with SMMs demonstrate progress in this research area since even though there are reports on the deposition of isolated molecules [18,116,121,123], the method of their separation has not been studied enough. The controlled deposition of SMMs with a defined level of separation is a challenging task that demands a specific synthesis route and chemical strategy. The modification of the synthesis procedure by using surface covering with specific functional groups can cause a substrate to become selective for certain molecules. For example, it is possible to control the distribution of Mn${}_{12}$ SMMs on the surface of silica spheres by using the 2D solid solvent method [215]. This approach also made it possible to estimate the influence of the substrate on the properties of the obtained nanocomposites [212,216], in particular to study the interactions between the anchored molecules, to study the interactions between the SMMs and the substrate, to analyze in detail how molecular magnetics are anchored, and to determine the induced changes in the symmetry of the molecule [115]. There is also a possibility of using spatial limitations of some nanostructures, like the inner space of carbon nanotubes, fullerenes, or pores of silica matrices or metal–organic frameworks. For example, the density of accommodated SMMs can be controlled by encapsulation into a single-walled carbon nanotube [176]. Another possibility for the controlled distribution of molecules is the use of well-organized pores of silica matrix (like SBA-15), which can provide an arrangement of molecules and possible inter-cluster distance control [50,189,190]. The controlled incorporation of SMMs into porous metal–organic frameworks was also reported [203,206]. The application of the MOF structure with well-ordered cavities provides the long-range nanostructuring of magnetic molecules, paving the way for new advanced nanodevices fabrication [205].

**Figure 6:**
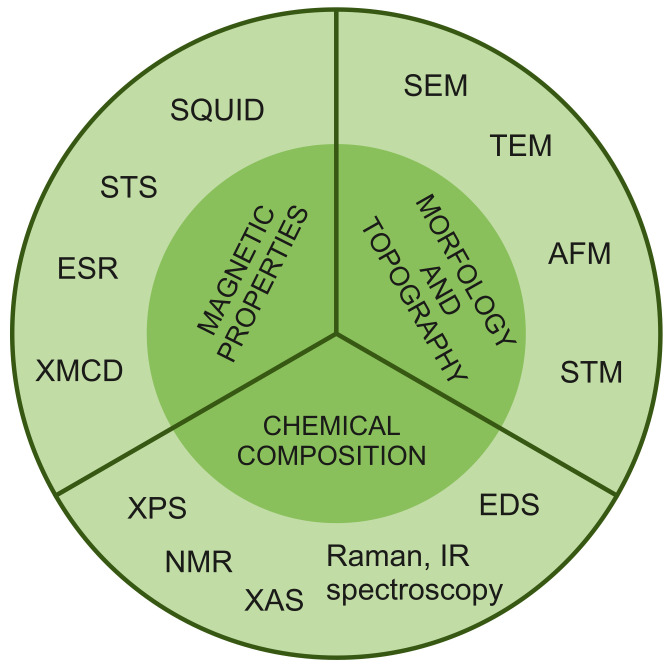
Methods of SMM/nanostructure composite characterisation.

Another important point in SMM/nanostructure composites investigation often relies on the necessity of using a combination of advanced and sophisticated imaging, spectroscopic, and magnetic property experimental techniques for the systematic study of the material (Figure 6). The reviewed papers show that curved surfaces (as in the case of NPs or carbon nanotubes) can be more advantegeous than flat supports. Because of the higher specific surface area of such substrates, they can accommodate substantially larger numbers of SMMs. This results in a much wider spectrum of experimental techniques for the investigation and even observation of individual molecules without using sophisticated techniques, such as synchrotron radiation measurements (i.e., XAS, XMCD) or STM microscopy. The use of spherical geometry of silica, for example, made it possible to observe Mn_12_-core molecules located at the horizon of the sphere by transmission electron microscopy [217]. The observation of Fe_4_ molecules attached to the surface using the TEM technique was also reported for the case of MWCNTs [167]. Using the same substrate, the transmission electron microscopy images of encapsulated Mn_12_-ac molecules individual clusters were presented as well [177]. Thus, there is a possibility to confirm the effective immobilization of SMM on the surface of the substrate with conventional microscopic research techniques using the proper substrate and type of SMM. However, very often the observation of deposited molecules, especially on flat surfaces, requires employing advanced techniques, such as the surface magneto-optical Kerr effect measurements, or spin-polarized STM or MFM. Particular examples show that magnetic molecules have a trend to form aggregates, closely packed arrays, or monolayers while deposited on the flat substrate (i.e., Au [162] or MgO [198] thin films, graphene [129,180,182]). Therefore, the evidence of their presence and the method of distribution on the substrate demands the application of high-resolution scanning transmission microscopy.

Concerning the applications, nanocomposites consisting of an SMM and a neutral matrix are mainly intended for use in nanoelectronics. However, as it was shown, the way from the synthesis of SMMs containing nanocomposite to its practical use in various industrial fields demands solving numerous issues such as the confirmation of the effective attachment of molecular magnetics on the surface, the control of their distribution, and the preservation of their magnetic properties. Particular interest here is focused on the type of substrate. For example, electrically conducting substrates offer a great platform for integrating SMMs into electronic and spintronic devices. A combination of their magnetic properties with the plasmonic and transport properties of gold, for example, can give a multifunctional hybrid material [122]. The slow magnetization relaxation and large magnetic anisotropy of molecular nanomagnets’ can be exploited by depositing them on such substrates, thus allowing for the design of novel devices such as spin-based transistors [218], magnetic memory elements [65], and quantum computing components [219,220]. The conducting substrate provides the necessary electrical connections and enables the incorporation of single-molecule magnets into practical electronic circuits [120]. Also, by depositing multiple SMMs on the substrate in close proximity, one can create coupled systems that exhibit collective magnetic behavior. The conducting substrate acts as a mediator, allowing for magnetic interactions between adjacent SMMs, leading to phenomena such as magnetic ordering, spin dynamics [221], and quantum entanglement [222], which are of great interest for both fundamental research and potential technological applications. All of these promising features incline researchers to investigate SMMs anchored on electrically conducting platforms and explore their behavior not only on gold but also on nanostructured carbon (i.e., graphene, carbon nanotubes, fullerenes, and their derivatives), which is well known for possessing outstanding electrical transport properties, arising mainly because of the monolayer hexagonal arrangement of the carbon atoms, and their sp^2^ hybridization [223]. The electrical conductivity of the carbon nanotubes, for example, which can be accurately regulated by the magnetic states of SMMs, can provide a mechanism for creating spin-polarized currents at the nanoscale, which is significant for the field of spintronic devices [177]. The combination of SMMs with graphene, on the other hand, can enable electrical spin manipulation and control, which opens the way for novel nanoelectronic devices [180,213].

Special interest is also attributed to non-conducting substrates as the platforms for SMMs immobilization. Nanostructured forms of silica, for example, are an excellent materials as a substrate, due to their diamagnetic properties, their thermal and mechanical stability, and an almost unlimited ability to anchor various chemical functional groups on their surface. Using silica nanoparticles or mesoporous silica matrices containing magnetic molecules seems somewhat abstract but has a very strong application translation. The use of silica spheres, for example, allowed one to confirm the effectiveness of the synthesis methods and also to gather basic knowledge about the behavior of Mn_12_-core molecules on silica surfaces [114]. Moreover, synthesis procedures applied can be adapted to the functionalization of suitable thin-film materials obtaining systems of bistable magnetic units. It is theoretically possible even to arrange them two-dimensionally by using nanostructuring of the substrate [161]. Such structures, in turn, have great potential for application in nanoelectronics as super-dense memory storage devices, or elements of molecular neural networks [224,225].

Applying single-molecule magnets onto nanostructures frequently necessitates the refinement of synthesis methods, a combination of advanced measurement techniques, and the revealing of their specific characteristics for particular applications. This field, however, not only serves to advance our fundamental understanding of nanomagnetism but also opens the way for the development of innovative technologies that rely on the distinct properties of SMMs on nanostructured platforms. Undoubtedly, such composites may serve as components of innovative nanoelectronic and spintronic devices—spin valves, spin transistors, units of ultrahigh-density magnetic memory, magnetic sensors, qubits, or spin-based logic gates for quantum computing. Obtaining such devices, however, will require further investigation in the field of molecular magnetism, especially expanding our knowledge regarding the interactions occurring between magnetic molecules and their host systems.

## 5. Summary

In this review article, the deposition of single-molecule magnets on nanostructures is examined, providing a broad overview of key aspects of the field. Beginning with an introduction, the importance of this research area and its potential for the development of magnetic materials at the nanoscale is highlighted.

Based on the synthesis procedures and characterization of the main chemical classes of SMMs, their unique magnetic properties are discussed and notable examples are highlighted. In addition, the possibility of altering the outer organic shell of SMMs is shown, and the particular importance of such a process in anchoring molecules to the substrate and modifying their magnetic properties is pointed out. Most importantly, arguments are emphasized for the idea of nanostructures as suitable platforms for SMMs deposition, noting both the nature of the substrate and its dimensionality. This approach makes it possible to identify the most important characteristics with which the substrate groups are characterized, as well as to indicate their influence on the resulting SMM/substrate complexes. Firstly, nanometer structures are characterized by a high surface-to-volume ratio, which allows for a higher density of nanomagnet deposition, increasing the chances of effectively measuring the system’s response to external stimuli. Moreover, it is possible to engineer and adjust the available surface by controlling the shape, size, and properties of given nanostructures during the synthesis stage. Finally, by selecting appropriate nanostructures, it is possible to modulate the interaction between the SMM and the substrate or host system. Such interactions, resulting from electrical conductivity or topological properties, can affect magnetic properties such as magnetic anisotropy and relaxation dynamics.

The process of attaching SMMs to substrates is one of the key steps in the field of nanomagnets research (see: Section 4). Many of the works cited focus on the selection of the synthesis method, the proper preparation of substrates, and the development of a methodology to confirm the success of the procedure. It has been shown that, due to the complex characteristics of SMMs and their diverse interactions with various nanostructures, a much deeper analysis and more comprehensive study of their composites is necessary. Such an analysis warrants an understanding of how the molecules are deposited on the surface. Molecules can be immobilized by non-specific van der Waals forces or specific chemical interactions that cause significant changes in electron states. In addition, interactions may involve non-covalent π-π stacking or encapsulation in the internal spaces of nanostructures. All of these define the properties of the final composite material and determine its applicability.

The low-dimensional substrates offer a multifaceted advantage by not only ensuring the controlled deposition of molecules with the probable possibility of controlling their distribution on the surface but also providing the preservation of their unique magnetic characteristics. What is more, the proper selection of a nano-sized substrate, combined with a tailored deposition methodology, can yield enhancements in the magnetic properties of SMMs, such as coercive field, relaxation times, and blocking temperature. Employing low-dimensional substrates as platforms for the deposition of single-molecule magnets provides unique nanocomposite structures, which can meet the specific requirements of diverse applications driving innovation and enabling groundbreaking advancements in the field of nanomagnetism and quantum technologies.

However, for these advancements to come true, significant efforts must be directed toward specific key development routes.

First of all, the search for nanomaterials with a high surface-to-volume ratio must continue. Then, the necessity for a proper understanding of SMM-substrate interactions must be met, both theoretically and empirically. This, in turn, requires constant improvement and increased accessibility of our experimental methods. For example, STM imaging proved to be a very useful tool in recent years for comprehending the deposition of molecules on surfaces. To broaden access, particularly for the low-temperature imaging of complex magnetic molecules, efforts are needed. AFM with functionalized tips shows promise, offering wider accessibility than STM and overcoming substrate conductivity requirements. AFM is not affected by diamagnetic decoupling layers, such as MgO or organic molecules, making it versatile. Current research focuses on efficiently decoupling magnetic molecules from surface phonons and achieving extensive, homogeneously oriented coverage, wich is critical for advancing molecule-based electronics and information storage. At present, researchers are exploring a plethora of novel single-molecule magnets and various types of nanostructures. Testing the myriad combinations is necessary to identify the most optimal and advantageous ones, which is a process that is undoubtedly neither easy nor quick. In recent years, a considerable amount of research is dedicated to (among others) the Mn_6_ sub-family [42,226,227]. This trend is expected to persist, given the adaptability of these species for ligand exchange and property tuning. Also, as mentioned in Section 3, YIG matrices show potential for coupling deposited SMMs and facilitating communication between them, bringing us closer to constructing fully operational, SMM-based nanodevices. On the other hand, metallocene dysprosium SIMs have, in recent years, enabled the achievement of blocking temperatures surpassing the ’magical’ liquid-nitrogen level. Consequently, it would be compelling to couple them with a preferred type of nanostructure, especially considering that studies have already demonstrated such coupling in the case of metallocene-SWCNT hybrids [228]. Here, the latest achievements by Komeda and co-workers, concerning the employment of superconducting NbSe${}_{2}$ substrates for TbPc${}_{2}$ molecules, are also worth mentioning, although the authors did not include information about the thickness of the used NbSe${}_{2}$ layer, which made it hard for us to consider it a nanostructure [229,230]. Nevertheless, the coupling of superconducting materials and SMMs seems to be a very appealing and promising route, the exploration of which should be continued.

In the coming years, advancements in imaging and the organization of magnetic molecules on surfaces, while retaining their intended magnetic properties, will be at the forefront of discussions. Positive strides in these areas could pave the way for collaborative efforts involving synthetic chemists, physicists, and engineers, ultimately culminating in the realization of anticipated molecular-based devices that contribute to more efficient, faster, and lower-energy technology.

## Figures and Tables

**Table 1:** The list of exemplary molecular nanomagnets, including the most popular ones, along with their basic properties. We denote **S_T_** and **Δ/k_B_** as the total ground-state spin value and the magnetization reversal energy barrier, respectively.

SMM	Magnetic Core	Ligands	S_T_	Δ/k_B_	T${}_{B}$	Literature
**Mn_12_-ac**	4 × Mn^4+^	(CH_3_COO)${}_{16}$	10	65 K	3.0 K	[5,26]
	8 × Mn^3+^					
**Mn_12_-benzoate-ADC**	4 × Mn^4+^	(O_2_CPh)_8_(ADC)${}_{4}$	10	65.2 K	4.0 K	[45]
	8 × Mn^3+^					
**Mn_12_-betHPF_6_**	4 × Mn^4+^	[(bet)_16_(EtOH)_4_](PF_6_)14·4CH_3_CN	11	34 K	2.0 K	[49]
	4 × Mn^3+^					
	4 × Mn^2.5+^					
**Mn_12_-phn**	4 × Mn^4+^	(O_2_PC_12_H_10_)_12_(O_2_CCH_3_)_4_	10	49 K	3.2 K	[33]
	8 × Mn^3+^					
**Mn_12_-st**	4 × Mn^4+^	(CH_3_(CH_2_)${}_{16}$CO_2_)${}_{16}$	10	62 K	2.75 K	[31,50]
	8 × Mn^3+^					
**Mn_12_-oxocarb**	4 × Mn^4+^	(CN-o-C_6_H_4_CO_2_)_12_(CH_3_CO_2_)_4_	10	58.7 K	4.2 K	[102]
	8 × Mn^3+^					
**Mn^III^_6_O_2_**	6 × Mn^3+^	(Etsao)_6_O_2_CPh(Me)_2_(EtOH)_6_	12	86.4 K	4.5 K	[103]
**Fe_4_**	4 × Fe^3+^	(CH_3_O)_6_(dpm)_6_	5	7 K	1.0 K	[51]
**Fe_8_**	8 × Fe^3+^	(OH)_12_(tacn)_6_	10	22.2 K	3.0 K	[53,57]
**Fe_22_**	22 × Fe^3+^	(OH)_3_(O_2_CMe)_21_(mda)_6_	0	-	-	[62]
**Fe_4_(L^1^)_2_(dpm)_6_**	4 × Fe^3+^	(L^1^)_2_(dpm)_6_ *	5	15.9 K	-	[63]
**Fe_4_(acac)_6_(Br-mp)_2_**	4 × Fe^3+^	(acac)_6_(Br-mp)_2_	5	17.3 K	-	[69]
**Ni_24_**	24 × Ni^2+^	(O_2_CMe)_42_(mdaH)_6_(EtOH)_6_	6	0.24 K	-	[62]
**Cr_7_Ni**	7 × Cr^3+^	(^t^BuCOO)_16_	0.5	-	-	[76,77,78]
	1 × Ni^2+^					
**TbPc_2_**	Tb^3+^	C_32_H_18_N_8_	6	740 K	∼1.0 K	[86,104]
**[Tb[Pc(OEt)_8_]_2_][SbCl_6_]**	Tb^3+^	[Pc(OEt)_8_]_2_][SbCl_6_]	6	791 K	-	[105]
**[TbPc_2_]/[Bu_4_N][Br]**	Tb^3+^	Bu_4_N][Br]	6	922 K	-	[106]
**[Dy(acac)_3_(H_2_O)_2_]**	Dy^3+^	(acac)_3_(H_2_O)_2_	6	67.3	∼2.0 K	[107]

* L^1^—2,2-bis(hydroxymethyl)-10-undecen-1-ol. Other abbreviations may be found in the Abbreviations section.

## Data Availability

Data are contained within the article.

## Abbreviations

**SMM**	single-molecule magnet
**ZFS**	zero-field splitting
**QTM**	quantum tunneling of magnetization
**TAQTM**	thermally activated quantum tunneling of magnetization
**ITO**	irreducible tensor operators
**Cp^iPr5^**	penta-iso-propylcyclopentadienyl
**Cp***	pentamethylcyclopentadienyl
**ac**	acetate
**st**	stearate
**bet**	betaine
**phn**	diphenylphosphinate
**sao**	salicylaldoxime
**acac**	acetyloacetone
**dpm**	dipivaloylmethane
**tacn**	triazacyclononane
**Br-mp**	2-(bromomethyl)-2-(hydroxymethyl)-1,3-propanediol)
**SOC**	spin–orbit coupling
**DC**	direct current
**AC**	alternating current
**EPR**	electron paramagnetic resonance
**AFM**	atomic force microscopy
**ESR**	electron spin resonance
**XPS**	X-ray photoelectron spectroscopy
**NMR**	nuclear magnetic resonance
**EDS**	energy-dispersive X-ray spectroscopy
**SEM**	scanning electron microscopy
**TEM**	transmission electron microscopy
**SIM**	single-ion magnet
**SWCNT**	single-walled carbon nanotube
**MWCNT**	multi-walled carbon nanotube
**MOF**	metal–organic framework
**SQUID**	superconducting quantum interference device
**NP**	nanoparticle
**IR**	infrared
**HRTEM**	high-resolution transmission electron microscopy
**XANES**	X-ray absorption near edge structure
**HDA**	hexadecylamine
**mda**	methyldiethanolamine
**XMCD**	X-ray magnetic circular dichroism
**STM**	scanning tunnelling microscopy
**CVD**	chemical vapor deposition
**DFT**	density functional theory
**STS**	scanning tunnelling spectroscopy
**NEMS**	nanoelectromechanical system
**CNT**	carbon nanotube
**GMWCNT**	graphitized multi-walled carbon nanotube
**scCO_2_**	supercritical CO_2_
**DCE**	dichloroethane
**FET**	field-effect transistor
**POM**	polyoxometalate
**XAS**	X-ray absorption spectroscopy
**SAM**	self-assembled monolayer
**HOPG**	highly-oriented pyrolytic graphite
**NO**	nitric oxide
